# Complexation of CcmB with CcmACD safeguards heme translocation for cytochrome *c* maturation

**DOI:** 10.1002/mlf2.12150

**Published:** 2025-01-06

**Authors:** Yuanyou Xu, Wei Wang, Qianrou Zhang, Sirui Han, Jiahao Wang, Shihua Wu, Haichun Gao

**Affiliations:** ^1^ Institute of Microbiology and College of Life Sciences Zhejiang University Hangzhou China

**Keywords:** CcmABCD, cytochrome *c*, cytochrome *c* biosynthesis, heme, heme translocation

## Abstract

Cytochrome *c* maturation (CCM), a posttranslational modification involving covalent attachment of heme to polypeptides (apocyt *c*), is essential for the activity and cellular function of cytochromes *c*. Here, we identify and substantiate CcmB as heme translocase in bacteria. When in excess, CcmB expels intracellular heme into the periplasm and thus is detrimental to the cell. We then show that complexation with CcmACD ensures heme translocated by CcmB to be used for CCM only. Moreover, structural analysis and atomistic molecular dynamics simulations reveal that CcmB absorbs heme from the membrane to a heme pocket formed in the dimer interface of the transmembrane helix‐bundles. These data, collectively by providing detailed insights into the conformational landscape of CcmB during heme entry, fill in the missing link in our understanding of the heme translocation for CCM.

## Impact statement

Cytochromes *c* are essential to diverse biological processes, including respiration and photosynthesis. Among components of System I cytochrome *c* maturation (CCM) machinery, CcmB is the only one that remains functionally undefined. Here, we uncover that CcmB functions solely as heme translocase, expelling heme from the membrane to the periplasm, but its complexation with CcmACD ensures heme to be used for CCM exclusively. Our findings not only solve a decade‐long mystery regarding heme transport across the bacterial membrane for CCM but also open an avenue for the secretory production of free heme.

## INTRODUCTION

Hemes, iron‐bound porphyrins essential to all living organisms, are composed of protoporphyrin IX (heme *b*) and its derivatives, such as heme *a*, *d*, and *o* ^1^, ^2^. As the most versatile and ubiquitous metalloprosthetic factor, hemes endow hemoproteins to play irreplaceable roles in a myriad of physiological processes, including respiration, photosynthesis, oxygen transport, electron transfer, oxidative stress response, and catalysis^1^, ^2^. While a large number of hemoproteins contain heme *b* (or one of its derivatives) that is bound noncovalently, cytochromes *c* (cyts *c*) carry one or more heme *b* molecule(s) through covalent linkages^3^. Formation of such a linkage between apocyt *c* and heme is called cyt *c* maturation (CCM), which can be catalyzed by one of three enzymatic systems: System I (CcmABCDEFGH(I)), System II (CcsBA), and System III (HCCS), depending on species^3^.

**Figure 1:**
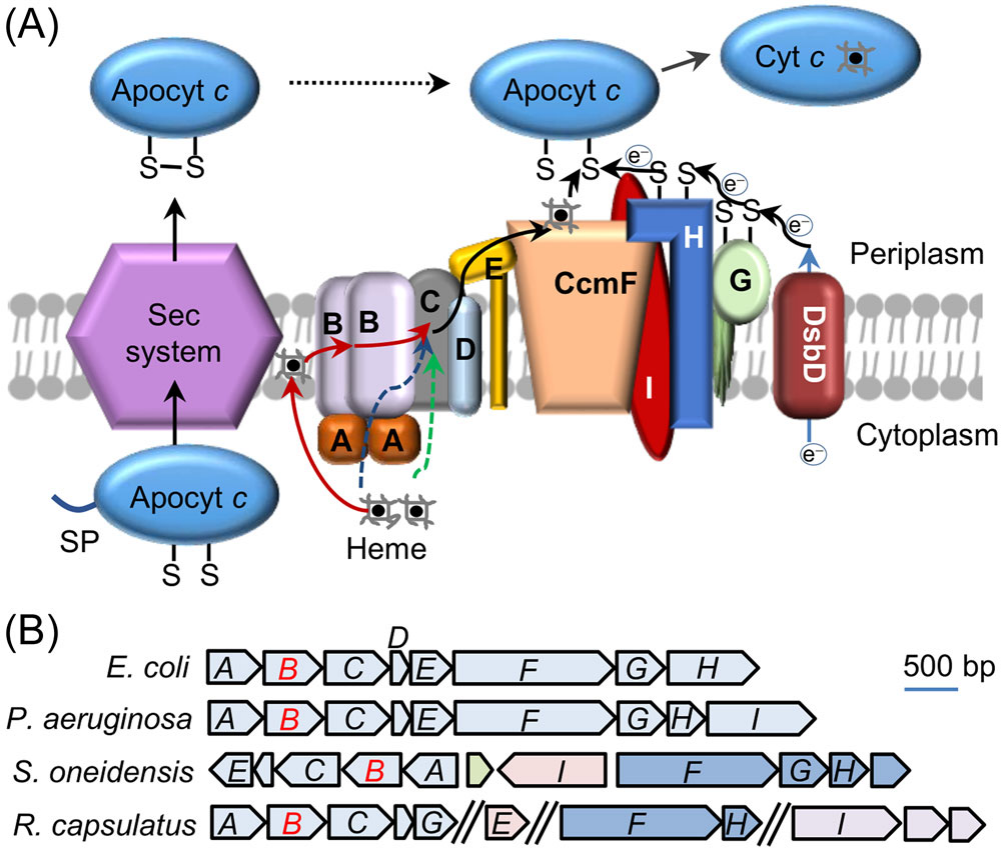
Schematic overview of System I cyt *c* maturation machinery (CCM). (A) The components of System I can be grouped into two modules, CcmABCDE for heme translocation and CcmFGH(I) for ligation of heme to apocyt *c*. After entering the periplasm via the Sec system, apocyt *c* is oxidized first and then reduced by electrons through an electron transfer relay involving CcmGH(I) and DsbD, which gets electrons from the cytoplasm. CcmE delivers heme from CcmABCD to CcmF, depending on ATP hydrolysis catalyzed by CcmA. Before this study, it has been proposed that heme is translocated across the membrane by CcmC (green dash line). Our data presented here conclude that heme is translocated by CcmB from the membrane to CcmC (red solid line). Whether CcmB is able to translocate heme from the cytoplasm to CcmC directly remains unknown, but this route clearly plays a minor role (blue dashed line). (B) Organization of *ccm* genes in representative bacteria. Genes in the same operon are backgrounded in the same color. Genes other than *ccm* are not labeled. Genes not next to each other on the chromosome are separated by //.

System I complexes, widely distributed in diverse Gram‐negative bacteria and archaea, as well as in plant and protozoan mitochondria, are typically composed of 8 or 9 proteins (CcmA‐H or CcmA‐I)^3^ (Figure 1A). These components can be organized into at least two functional modules, CcmABCDE and CcmFGH(I)^4^ (Figure 1B). The CcmABCDE module comprises the CcmABCD complex, which is responsible for heme translocation across the cytoplasmic membrane, and CcmE, which serves as a periplasmic chaperone to receive and deliver the heme molecule to the CcmFGH(I) module for heme ligation to apocyt *c* ^5^, ^6^, ^7^.

It is now clear that CcmABCD constitutes an ABC transporter specialized for heme translocation across the cytoplasmic membrane, but the composition of the complex is not certain yet: two (AB)_2_CD and (ABCD)_2_ complexes have been reported most recently based on AlphaFold2 (AF2) Complex prediction and cryo‐EM structures^8^, ^9^, ^10^. Nevertheless, in this ABC system, CcmA is undoubtedly a classic ATP‐binding cassette protein in the cytoplasm, which binds and hydrolyzes ATP to drive conformational changes in integral membrane proteins CcmB and CcmC^9^. Because CcmC was found to be not only essential but also sufficient (when in excess) for heme transfer and attachment to CcmE, it has been proposed to be the heme‐translocating component^11^. This notion is strengthened by the fact that CcmC harbors the heme‐binding site, a conserved tryptophan‐rich region, termed the WxWD domain as found in System II heme transporter CcsBA^2^, ^12^. CcmD, a small single transmembrane (TM) polypeptide, is bound to CcmC only to presumably stabilize the ABC complex, and consistent with this role it is required for the release of holo‐CcmE from CcmC but not for heme translocation^13^.

Although CcmB is essential for CCM, the exact role that CcmB plays has been enigmatic for decades^14^. This becomes more unbearably obscure as recent structure illustrations of the CcmABCD complex have failed to provide valuable insights into heme translocation across the membrane^9^, ^10^. In this study, we solved this mystery by using *Shewanella oneidensis* as the research model. *S. oneidensis* encodes a large repertoire of cyts *c* (at least 41), which confer on cells (colony and cell‐pellet) a red‐brown color. As the abundance of cyts *c* correlates well with the color intensity, this phenotypic signature can be exploited to screen genes associated with changes in the cyt *c* content^4^, ^15^, ^16^, ^17^, ^18^, ^19^. With a whole‐genome expression library, we identified CcmB as a protein that exports heme when it is overproduced. This activity was found to be conserved among CcmB proteins in bacteria hosting System I. Importantly, the complexation of CcmB with CcmACD directs heme to be used for CCM exclusively. We then showed that the heme translocated by CcmB is from the cytoplasmic membrane. Furthermore, combined with molecular modeling, residue dynamic calculations, and mutational analysis, we identified a pocket within CcmB for heme and a heme entry from the membrane. By illustrating CcmB as a heme translocase, the missing link in CCM, the presented study provides insights into the mechanisms that govern heme translocation across the membrane.

## RESULTS

### Excess CcmB reduces the cyt *c* content and impairs growth in *S. oneidensis*

With *S. oneidensis* as a model, prior attempts with transposon mutagenesis identified many genes in their absence, and overproduction in rare cases, which impact the cyt *c* content by screening for mutants that have a noticeable difference in colony color^15^, ^16^, ^17^, ^18^, ^19^, ^20^, ^21^. However, these genes, accounting for a vast majority of color‐different mutants obtained to date, had been repeatedly caught in each round of screening, rendering the method no longer effective for identifying new genes that regulate the cyt *c* content when expressed at altered levels.

**Figure 2:**
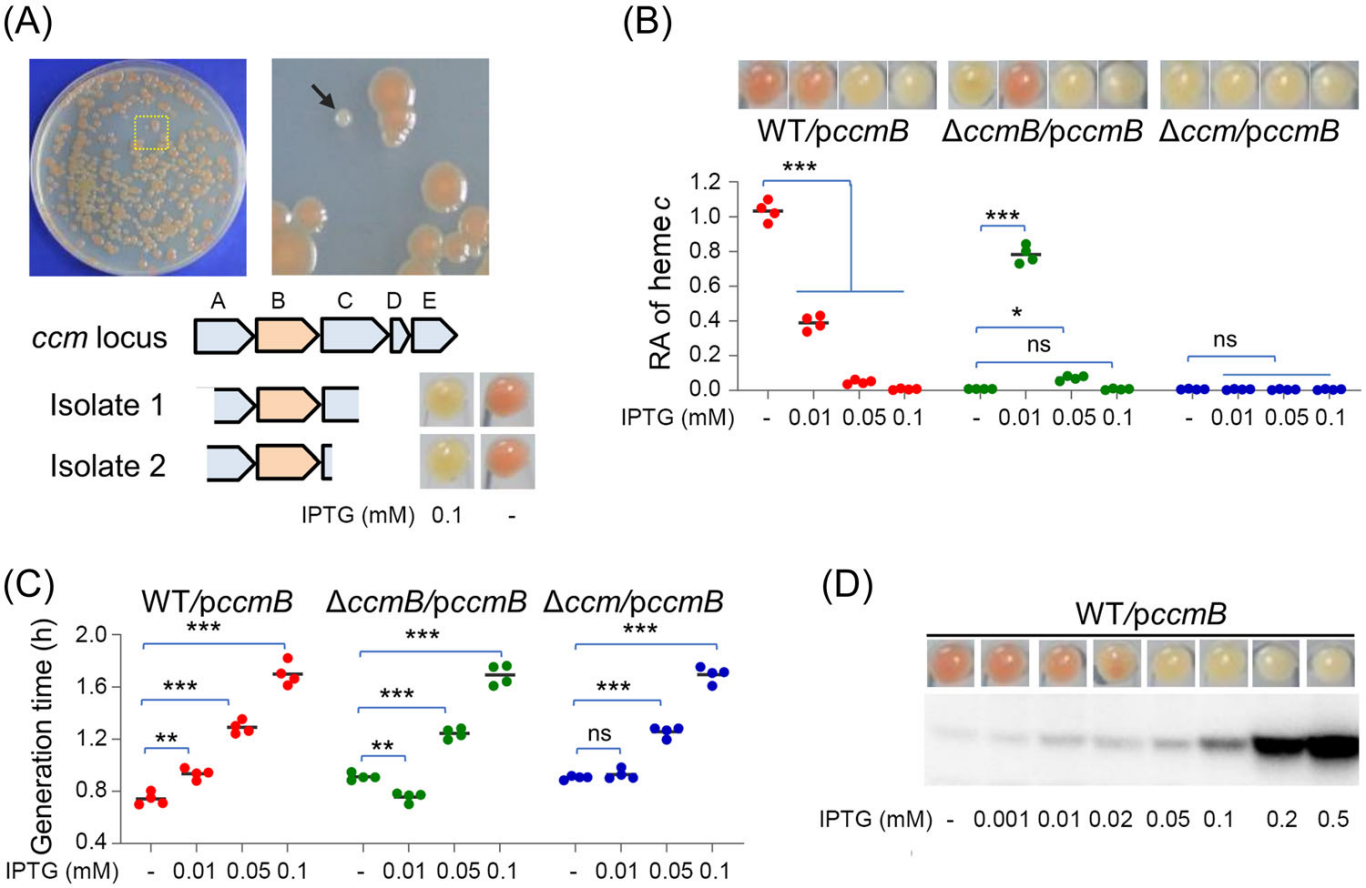
Identification of *ccmB* whose overexpression impairs CCM. (A) Screening for mutants having altered cyt *c* contents. A whole‐genome library was expressed under IPTG‐inducible promoter P_*tac*_ in *Shewanella oneidensis* wild‐type (WT) with IPTG at 0.1 and 0.5 mM. Shown is an isolate pointed by an arrow that lost its reddish‐brown color. The DNA fragments inserted into the vectors from two white‐colony isolates are given below. Without IPTG (−), these two isolates were not different from WT in color. (B) CcmB overexpression compromises CCM. All strains carried a vector expressing *ccmB* driven by P_*tac*_ (p*ccmB*). Shown are cell pellets of the early stationary phase cultures grown with IPTG at varying levels, which were then lysed for quantification of heme *c*. The obtained values were first adjusted to protein levels of samples, and then the averaged values for each strain were normalized to that of WT grown without IPTG, which was set to 1, giving to relative abundance (RA). (C) CcmB overexpression impairs growth. The growth dynamics are given in Figure S1B, from which generation time was calculated. (D) CcmB levels in WT carrying the expression vector that grew with IPTG at varying concentrations were assessed by western blot analysis using antibodies against His_6_‐tagged CcmB. In all panels requiring statistics analysis, for the values compared, ns, not significant; *p < 0.05; **p < 0.01; ***p < 0.001.

To circumvent this, an *S. oneidensis* genomic library on an expression vector driven by isopropyl‐β‐D‐thiogalactopyranoside (IPTG)‐inducible promoter P_*tac*_ was constructed and introduced into the wild‐type (WT) strain for differently colored colonies in the presence of 0.1 and 0.5 mM IPTG. With a total of ∼30,000 colonies screened for each IPTG concentration, we obtained two isolates that completely lost the reddish‐brown color from the plates containing 0.1 mM IPTG but not from those supplemented with 0.5 mM IPTG (Figure 2A). When grown in the absence of IPTG, these two isolates were not different from the parental WT with respect to colony color, suggesting that the phenotype is a result of the expression of the gene within the vector (Figure 2A). The genomic DNA fragments inserted into the vectors from these two isolates were found to cover the entire *ccmB* coding sequence as well as varying portions of the *ccmA* and *ccmC* genes, whose products would unlikely be functional (Figure 2A). To verify that CcmB is responsible for the phenotype, we cloned and expressed *ccmB* in WT, ∆*ccmB* (*ccmB* in‐frame deletion strain), and ∆*ccm* (devoid of the entire Ccm system). Because CcmB with His_6_‐tag at the C‐terminus behaved the same as the native protein in terms of their impacts on growth and CCM (Figure S1A), the subsequent experiments were carried out with this recombinant protein to facilitate detection unless otherwise noted. Heme *c* quantification revealed that the inhibitory effect of CcmB on the cyt *c* content increased with IPTG concentrations, with 0.1 mM being sufficient to completely abolish CCM in WT (Figure 2B). In addition, CcmB overproduction was found to be detrimental to growth, and this impact was also correlated to CcmB levels (Figures 2C,D and S1B). Notably, growth was barely visible for 24 h in the presence of 0.5 mM IPTG (Figure S1B), explaining why we failed to obtain CcmB‐overproducing isolates from the screening with 0.5 mM IPTG. Importantly, the cyt *c‐*deficient phenotype of ∆*ccmB* could be restored with IPTG at 0.01 mM, and further elevated expression reduced the cyt *c* content and inhibited growth (Figures 2B,C and S1B), supporting that all observations are due to altered expression of CcmB. The growth inhibition by overexpressed CcmB was also evident in ∆*ccm* (Figures 2C and S1B), manifesting that the effect is independent of a functioning System I CCM. Furthermore, we repeated the experiment with the *ccmC* gene and found that its expression with up to 0.5 mM IPTG, which fully restored CCM of a *ccmC* mutant, did not affect the cyt *c* content in WT (Figure S1C), indicating that CcmC could not function as CcmB. Altogether, these data substantiate that excessive CcmB has a detrimental impact on bacterial physiology, resulting in severe defects in growth and CCM.

### The effect resulting from excess CcmB is due to heme efflux

To unravel the mechanisms underlying the impact of excess CcmB, we first determined whether these CcmB proteins are correctly localized to the Inner membrane (IM) with CcmB‐GFP fusions. The fusion proteins were found to be active, capable of complementing the *ccmB* mutant and inhibiting CCM when expressed to proper levels (Figure S2A). Confocal laser scanning microscope observation showed that the overexpressed CcmB‐GFP in cells was able to localize in the membrane by comparing to the proteins known to be in the cytoplasm and IM‐bound (Figure S2B). Consistently, the results of western blot showed that CcmB‐GFP proteins were present in the membrane protein portion only (Figure S2B).

**Figure 3:**
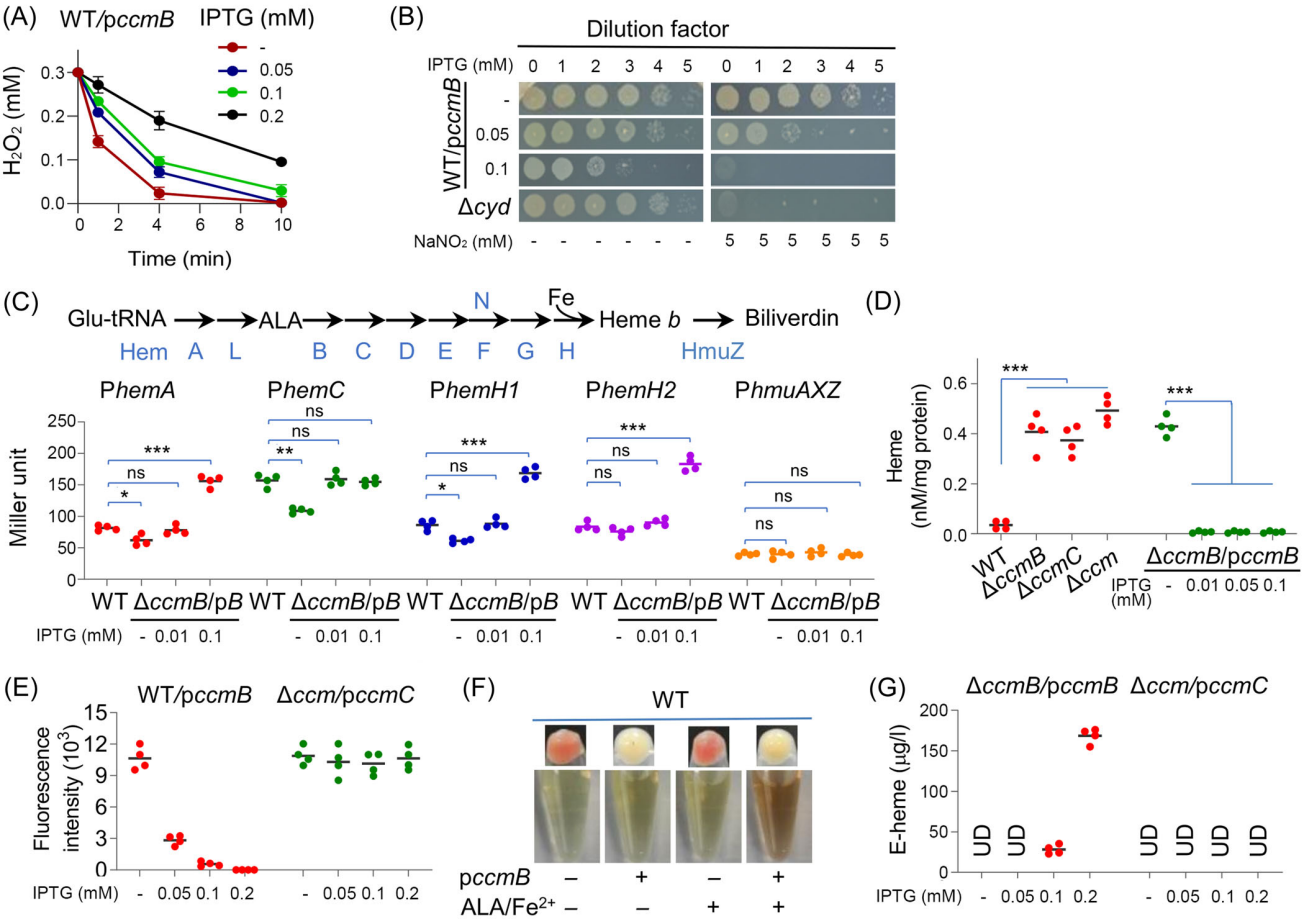
Overproduced CcmB causes heme efflux. (A) Catalase activity assay. H_2_O_2_ was added to mid‐exponential phase cultures to 0.3 mM, and concentrations of the remaining H_2_O_2_ after 1, 4, and 10 min were measured. The data of four replicates were presented as the mean ± SD. (B) Cyt *bd* activity assay. In *S. oneidensis*, cyt *bd* oxidase dictates nitrite susceptibility. Cultures at the mid‐exponential phase were adjusted to approximately 10^8^ CFU/ml (dilution factor, 0), followed by 10‐fold serial dilution, and 5 μl of each dilution was spotted onto the plates and growth was photographed 24 h later. Δ*cyd*, cyt *bd‐*deficient mutant. (C) Promoter activity assay of key heme biosynthetic and degradation genes. Pathways for heme biosynthesis and degradation in *S. oneidensis* are shown. The promoters under examination were placed before the full‐length *E. coli lacZ* gene within an integrative system. Cells grown to the mid‐exponential phase were collected and assayed for β‐galactosidase activity. Glu‐tRNA, glutamyl‐tRNA; p*B*, p*ccmB*. (D) Excess CcmB lowers intracellular heme concentrations. Cells grown to the mid‐exponential phase were pelleted by centrifugation, and heme content was determined on cell lysates. Heme content was normalized to the protein concentration. (E) Fluorescence of smURFP in strains carrying heme biosensor. Cells of the late‐exponential phase were pelleted by centrifugation and suspended in fresh medium, and fluorescence (642/670 nm) was measured after incubation at 4°C in the dark. (F) CcmB in excess effluxes heme. Pellets and supernatants of WT cells expressing *ccmB* grown in the absence or presence of 5 mg/l ALA and 1 mM FeSO_4_, and 0.1 mM IPTG. (G) Extracellular heme (E‐heme) accumulation. The pigment in the supernatants in (F) was determined by HPLC and quantified. UD, under detection limit. In all panels requiring statistics analysis, for the values compared, ns, not significant; *p < 0.05; **p < 0.01; ***p < 0.001.

Given that CcmB is an essential component of the heme‐translocating system, we hypothesized that the inhibition of CCM and growth by overproduced CcmB may be a consequence of lowered intracellular heme levels. To test this, intracellular heme concentrations were estimated with two heme‐dependent enzymes, cytoplasmic catalase KatB that dictates resistance to hydrogen peroxide (H_2_O_2_) and IM‐bound cyt *bd* oxidase that confers resistance to nitrite^22^, ^23^. Clearly, cells with excess CcmB exhibited substantially increased sensitivity to both H_2_O_2_ and nitrite (Figures 3A,B and S2C), supporting the compromised activity of these two enzymes, which is in accord with heme shortage.

To find an answer to lowered heme levels, we turned to heme biosynthesis and degradation. *S. oneidensis* employs the most common pathway for heme synthesis with glutamyl‐tRNA as the initial substrate and HmuZ as the primary enzyme for heme degradation^24^, ^25^ (Figure 3C). In the presence of overproduced CcmB, however, the expression of the genes for rate‐limiting enzymes in the heme biosynthetic pathways was found to be enhanced significantly (Figure 3C), suggesting an increased production of heme. In contrast, expression of the *hmuZ* gene was not affected (Figure 3C). Therefore, neither heme biosynthesis nor degradation is likely accountable for the detrimental effect of excess CcmB.

We then examined the possibility that the phenotype is due to heme export. To this end, the intracellular heme levels in ∆*ccmB* cells expressing *ccmB* to varying levels were assessed. By using both the QuantiChrom heme assay kit and the Turbo‐TMB (3,3′,5,5′‐tetramethylbenzidine) assay^26^, we found that intracellular heme contents could be confidently detected only in cyt *c‐*deficient mutants, such as ∆*ccmB*, ∆*ccmC*, and ∆*ccm*, which has been reported to have elevated heme levels^25^ (Figure 3D). In the presence of CcmB expressed with IPTG at as low as 0.01 mM, heme in cells was no longer detectable, supporting that CcmB exports heme (Figure S2D). To provide more confirmative results, we monitored heme levels with a hypersensitive heme biosensor exploiting small ultra‐red fluorescent protein (smURFP)^27^ (Figure S2D). In this biosensor, *Synechocystis* heme oxygenase (HO) was used to convert heme to biliverdin IXα, a heme‐degrading intermediate that can be covalently attached to smURFP in an autocatalytic manner, enabling far‐red‐light detection (Figure S2D). Coinciding with CcmB overexpression, *S. oneidensis* WT cells expressing HO with 0.2 mM IPTG lost the reddish‐brown color resulting from cyts *c*, apparently due to HO‐catalyzed heme degradation (Figure S2D). When smURFP was co‐expressed with HO, the cell pellets became slightly blue and far‐red fluorescence was visible under a confocal microscope (Figure S2D,E). This phenomenon was not observed with additional expression of CcmB (Figure S2D), suggesting that heme levels are too low to produce biliverdin IXα to levels sufficiently high to activate smURFP. Fluorescence measurements demonstrated that *ccmB* expression reduces intracellular levels of heme substantially with 0.05 mM IPTG, and fluorescence could not be detected with further enhanced expression (Figure 3E).

Extracellular heme levels were then assessed. The *ccmB* overexpression in WT with 0.1 mM IPTG did not evidently alter the color of the cultures/supernatants. However, when 5‐aminolevulinic acid (ALA) and FeSO_4_ were exogenously added, the supernatants became dark brown although the cell pellets remained white (Figure 3F). The pigment was then determined to be heme *b* and quantified. Results showed that the *ccmB* mutant did not release heme to levels above the detection limit but was able to do so when the *ccmB* gene was expressed with IPTG at 0.1 mM or above (Figure 3G). Moreover, we assayed the intracellular levels of heme in the WT with ALA and FeSO_4_ addition. However, the heme levels were found to be not significantly different from those in the non‐addition control (Figure S2F). In the end, we substantiated that CcmC is unable to expel heme to the periplasm by expressing CcmC to varying levels in ∆*ccm*, in which heme would be more likely to be released because of the lack of heme chaperone CcmE. However, the expression of *ccmC* in the presence of up to 1 mM IPTG did not cause significant reduction and increase in intracellular and extracellular heme concentrations, respectively (Figure 3E,G). Collectively, these results conclude that the increased CcmB production leads to heme efflux.

### Complexation of CcmB with CcmACD ensures heme translocated by CcmB to be used for CCM only

As an ABC transporter, the CcmABCD complex was suggested to translocate heme across the IM to deliver heme to CcmE in the periplasm more than three decades ago^28^. The very component responsible for the process was later proposed to be CcmC^11^. This notion, taken for granted even by two most recent studies illustrating structures of the CcmABCD complex^9^, ^10^ (Figure 1A), is clearly out of accord with our data presented above.

**Figure 4:**
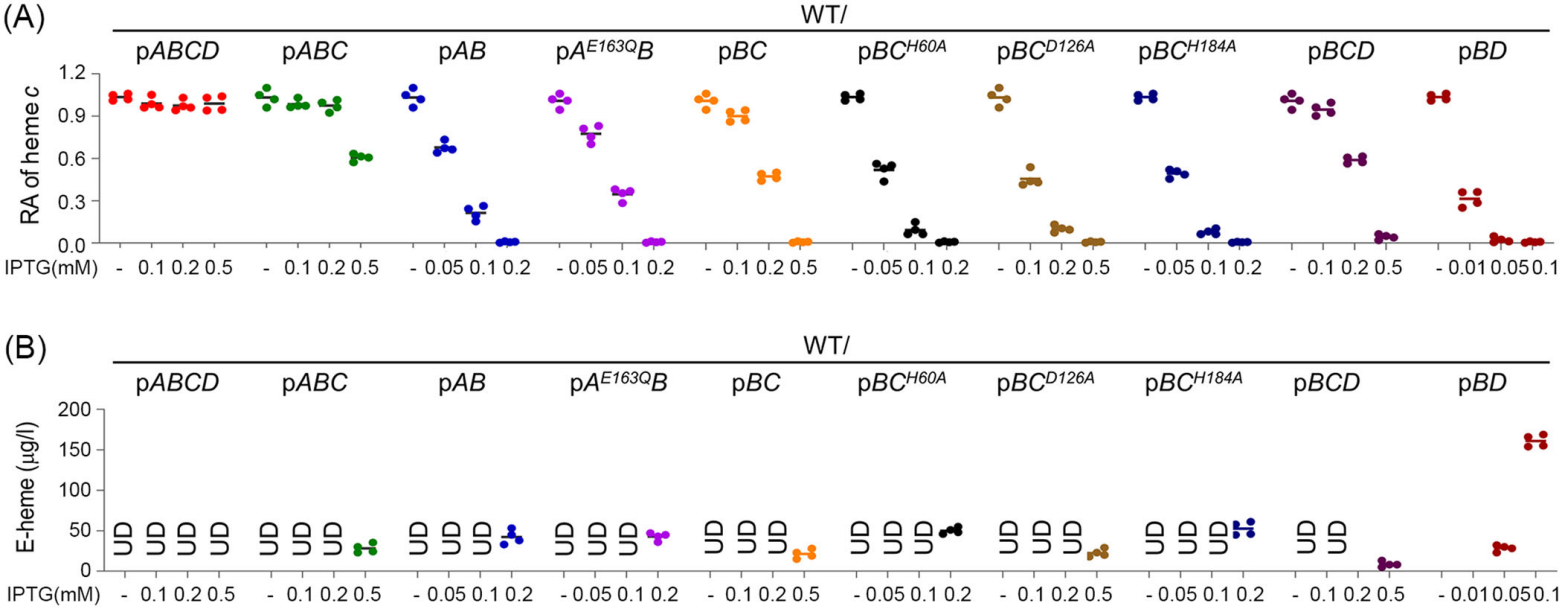
Complexation of CcmB with CcmACD safeguards heme translocated by CcmB to be used for CCM only. (A) The cyt *c* contents in WT expressing *ccmABCD* genes in varying combinations, including one CcmA and three CcmC variants. (B) E‐heme levels of the strains used in (A). Results of other CcmA and CcmC variants are given in Figure S3.

In attempts to solve this discrepancy, we assessed the influence of other components of the CcmABCDE module in varying combinations on CcmB‐mediated heme efflux. When the entire *ccmABCDE* operon or *ccmABCD* was expressed in WT with up to 0.5 mM IPTG, neither the cyt *c* content nor growth was significantly affected (Figures 4A,B and S3A), suggesting that the complexation of CcmB with CcmACD effectively prevents heme efflux caused by CcmB. However, the co‐expression of each of the other genes in this operon with *ccmB* failed to fully correct the observed defects (Figures 4A,B and S3A), stressing the significance of the whole complex. When overexpressed, CcmD alone (p*BD*) had no detectable antagonistic effect on CcmB‐mediated heme efflux, but its absence (p*ABC*) resulted in incomplete inhibition of heme export, presumably due to impaired stability of the ABC transporter complex. Both CcmC and CcmA significantly alleviated the heme‐efflux activity of CcmB, and clearly CcmC was more robust in counteraction (Figures 4A,B and S3A). Importantly, when expressed at proper levels, all of these combinations could fully restore CCM in respective mutant strains (Figures 4A,B and S3B).

The data presented thus far suggest that CcmB is the very component that translocates heme across the membrane and its complexation with CcmACD ensures translocated heme to be used for CCM. As CcmC is the component of the CcmABCD complex that binds and delivers heme to CcmE^5^, ^6^, ^7^, we hypothesized that CcmB‐mediated heme efflux would be affected if CcmC loses the ability to bind heme. To test this, we generated several CcmC variants, in which residues critical for heme binding (H60 and H184) or not (D126 and R128) are replaced by alanine^9^, ^29^. CcmB and each of these CcmC variants were then produced together to varying levels in the WT and ∆*ccmBC* strains. As expected, the CcmC variants (CcmC^D126A^ and CcmC^R128A^) that can complement the CCM defect of ∆*ccmBC* behaved the same as CcmC^WT^ (Figure S3B,C). In contrast, those lacking CcmC activity, CcmC^H60A^ and CcmC^H184A^, showed impaired ability to inhibit heme efflux (Figures 4A,B and S3A–C).

Based on the structure of the CcmABCD complex, a model has been proposed to suggest that Ccm(AB)_2_, the core ABC transporter, would undergo conformational changes, leading to heme release from CcmC^9^. We then tested if the conformational changes of Ccm(AB)_2_ would influence the heme efflux efficacy of CcmB. In *E. coli*, a CcmA E154Q variant locks Ccm(AB)_2_ to a fully closed configuration such that no conformational changes are allowed^10^. As this residue is perfectly conserved (Figure S3D, E163 in *S. oneidensis* CcmB), we generated CcmA^E163Q^ and other CcmA variants (CcmA^I41A^ and CcmA^L192A^) and assessed their impacts on CcmB‐mediated heme efflux. However, when co‐expressed with *ccmB*, none of these variants showed any detectable difference in the cyt *c* content, growth, or extracellular heme levels compared to the wild‐type CcmA, regardless of the presence of CcmA activity or not (Figures 4A,B and S3B,C), suggesting that the conformational changes of Ccm(AB)_2_ is irrelevant to CcmB‐mediated heme efflux. These data, collectively, conclude that CcmB alone has heme‐exporting activity and its complexation with other components to form the CcmABCD complex ensures the translocated heme is used for CCM only.

### Heme‐efflux activity of CcmB is highly conserved in bacteria hosting System I

**Figure 5:**
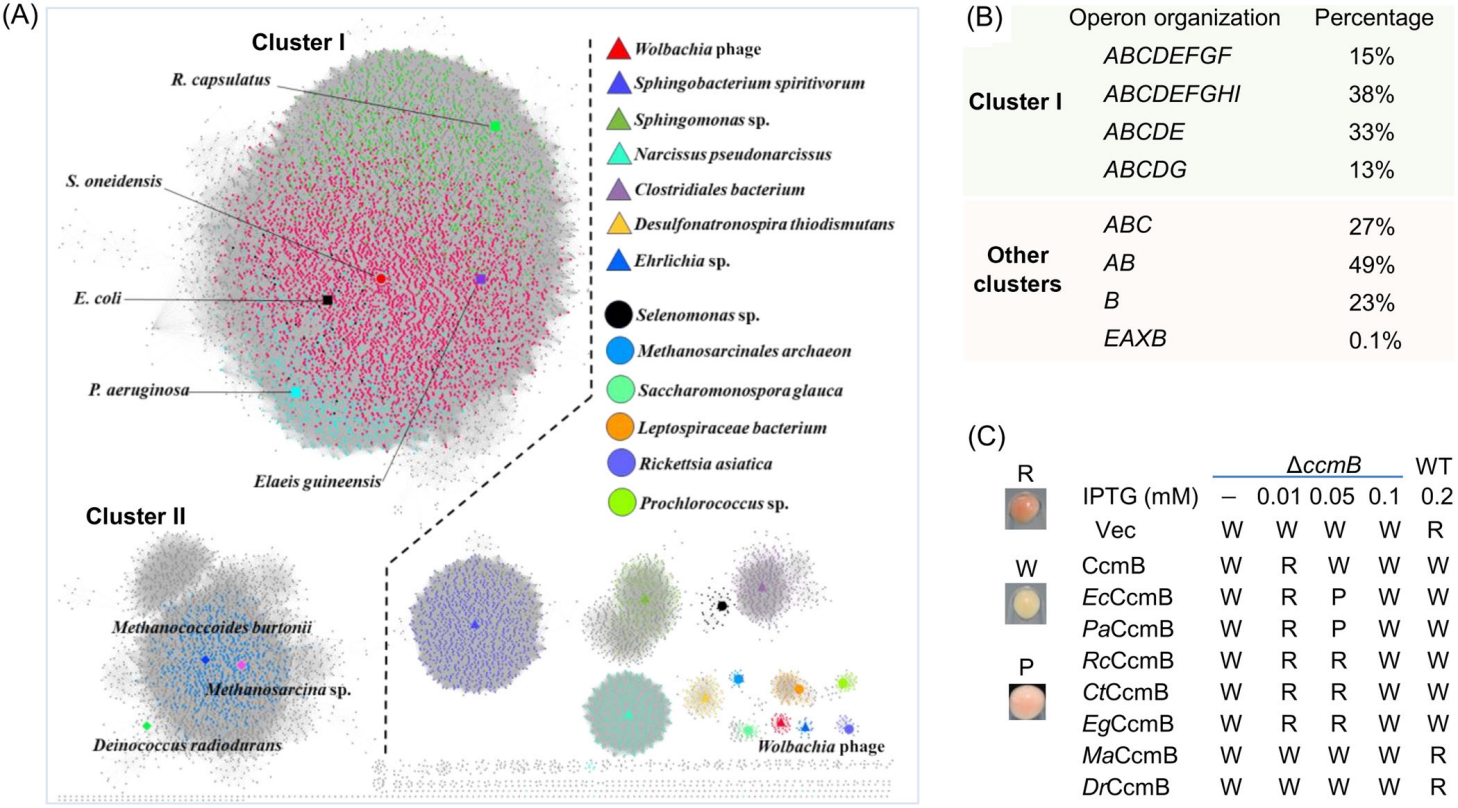
Heme‐efflux activity of CcmB is highly conserved in bacteria hosting System I. (A) A sequence similarity network (SSN) of CcmB homologs. The SSN was constructed by EFI‐EST with 2380 sequences from HMMER results with BLASTp E‐value of 1e−10 as the cutoff. The generated SSN reveals multiple clusters, in which each node represents a protein and is colored and shaped based on its classification. Species/strains of the same family are presented by the same color. CcmB homologs only from cluster I were found to be able to complement *S. oneidensis* CcmB (Table S1). (B) Diversity of the operons for CcmB homologs. Percentage represents each type of operon versus all operons. X represents the non‐ABC transporter protein. (C) The cyt *c* content of Δ*ccmB* or WT expressing one of representative CcmB homologs. Shown are the cell pellet color of cells expressing the genes of interest, W, whitish; R, reddish; and P, pinkish. Vec, empty vector. The cyt *c* content was quantified and presented in Figure S5A,B. *Ec*, *E. coli*; *Pa*, *Pseudomonas aeruginosa*; *Rc*, *Rhodobacter capsulatus*; *Ct*, *Comamonas terrigena*; *Eg*, *E. guineensis*; *Ma*, *Methanosarcina acetivorans*; and *Dr*, *Deinococcus radiodurans*.

To determine whether the heme‐efflux activity is a common feature of CcmB homologs, we analyzed CcmB proteins with multiple bioinformatics programs, including BLASTp, HMMER, EFI, and AF2 (Figure S4A,B). The results revealed that CcmB homologs from bacteria hosting System I constitute the main cluster (Cluster I) of a sequence similarity network (SSN)^30^ (Figure 5A). These CcmB homologs are found in two closely connected subgroups, represented by α‐ (green dots around *R. capsulatus*) and β/γ‐proteobacteria (purple and red/blue dots, respectively). Importantly, CcmB homologs of Cluster I are generally encoded by the genes within the *ccmABCD* operon (Figure 5B). On the contrary, the CcmB homologs in the organisms beyond α‐, γ‐, and β‐proteobacteria are grouped into distinct clusters and encoded by genes organized into diverse operons, suggesting weak evolution linkages among them (Figure 5A,B).

To test the heme‐efflux activity of CcmB homologs, we expressed five representatives from Cluster I in the Δ*ccmB* strain, including those from *E. coli*, *Pseudomonas aeruginosa*, *Rhodobacter capsulatus*, *Comamonas terrigena*, and *E. guineensis*. Not surprisingly, all of these proteins were able to complement the CcmB loss when expressed to proper levels and to inhibit CCM when overexpressed (Figures 5C and S4C–E), suggesting that these CcmB proteins efflux heme. However, it should be noted that *S. oneidensis* CcmB appeared more efficient than its homologs in exporting heme, such as the one of *E. coli* (Figures 5C and S4C–E). By comparison, representative CcmB homologs from other clusters, including *Dictyobacter kobayashii*, *Deinococcus radiodurans*, *Heava brasiliensis*, *Methanosarcina acetivorans* (archaea) and *Wolbachia* phage WO (virus), failed to function as *S. oneidensis* CcmB (Figures 5C and S4C–E; Table S1). Altogether, these data indicate that the heme‐exporting activity of CcmB homologs is conserved in bacteria hosting System I.

### CcmB exports heme primarily from the cytoplasmic membrane

Although it is clear that CcmB has an activity of heme efflux, a heme channel, via which heme travels across the IM, is present in CcsBA of System II but yet identified in CcmB^9^, ^10^, ^12^. To reconcile these conflicting observations, we envisioned that CcmB may translocate heme from the membrane to CcmC. It has been established that the cytoplasmic membrane is a primary target of exogenous heme and heme efflux systems expelling heme from the membrane, such as HrtAB of *Corynebacterium diphtheriae*, confer bacterial cells tolerance to heme toxicity^31^, ^32^, ^33^, ^34^. Therefore, we examined whether CcmB could function as HrtAB to detoxify exogenous heme.

**Figure 6:**
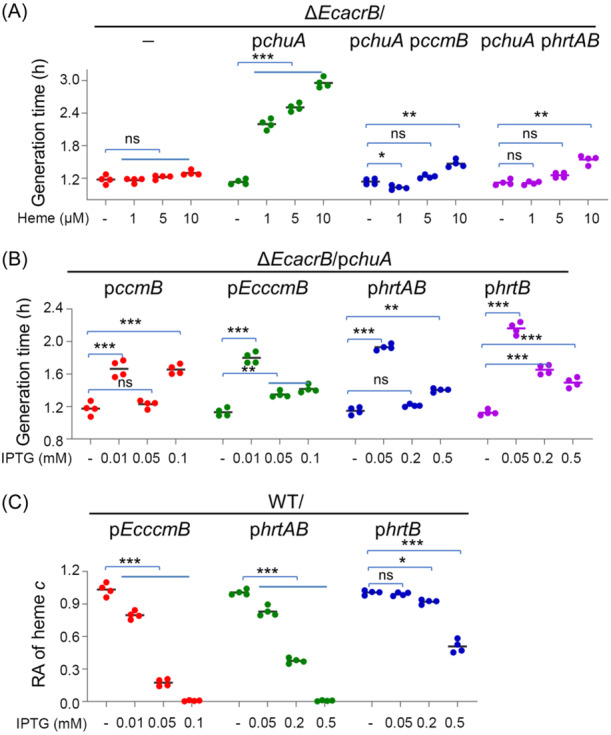
CcmB exports heme primarily from the cytoplasmic membrane. (A) Growth of heme‐resistant *E. coli*. Heme‐resistant Δ*EcacrB* could be sensitized to heme by expression of *chuA* driven by *arcA* promoter (refer to Figure S5). Expression of *ccmB* and *hrtAB* driven by P_*tac*_ was induced by 0.05 and 0.2 mM IPTG, respectively. (B) Growth rescue of heme‐sensitive *E. coli*. Heme‐sensitive Δ*EcacrB*/p*chuA* expressing each of the indicated genes to varying levels was grown with 5 μM heme. (C) The cyt *c* content of WT expressing each of the indicated genes to varying levels. In all panels requiring statistics analysis, for the values compared, ns, not significant; *p < 0.05; **p < 0.01; ***p < 0.001.

In *E. coli*, the loss of the dominant RND efflux pump component AcrB causes the outer membrane (OM) impermeable to heme and enhances resistance to exogenous heme whereas expression of ChuA, which transports heme into the periplasm, would substantially increase sensitivity to exogenous heme^34^, ^35^. An *E. coli acrB* null mutant (Δ*EcacrB*) was constructed and it grew similarly with heme supplemented in the range of 1 to 10 μM (generate time, ∼1.2 h) as previously reported^34^ (Figure 6A). When the *chuA* gene was expressed in Δ*EcacrB* driven by constitutively active but modest promoter for the *arcA* gene (P_*arcA*_), growth with heme in the same concentration range was significantly inhibited, with generation time substantially prolonged (minimum of 2.1 h) (Figures 6A and S5A), indicating that heme is cytotoxic to the cells once it passes through the OM. This growth inhibition was substantially alleviated when *S. oneidensis ccmB* was expressed with 0.05 mM IPTG in these cells (Figure 6A). However, with IPTG at 0.1 mM, growth was found to be compromised again, apparently due to heme efflux (Figure 6B). Similar results were obtained from *E. coli* CcmB expressed from the same expression system, whose expression was comparable to the *S. oneidensis* counterpart (Figures 6B and S4E).

In addition, the *hrtAB* operon of *C. diphtheriae* was cloned and expressed in the heme‐sensitive cells. Consistent with the finding reported before^34^, the expression with 0.2 mM IPTG largely relieved growth inhibition of heme (Figure 6A,B). Intriguingly, we found that when strongly induced, HrtB alone was also able to rescue growth, albeit less effectively than HrtAB (Figure 6B). Thus, all of these data are in line with the proposal that CcmB expels heme from the membrane as HrtAB does. Moreover, we expressed the *C. diphtheriae hrtAB* operon in *S. oneidensis*. HrtAB expression impaired CCM with 0.2 mM IPTG, but further enhanced induction was needed to cause significant growth inhibition (Figure 6A–C), suggesting that CcmB is more robust than HrtAB in expelling heme from the membrane. Expectedly, HrtB alone was less effective than HrtAB in reducing the cyt *c* content in *S. oneidensis* (Figure 6C). Altogether, we conclude that the heme translocated by CcmB is primarily, if not exclusively, from the cytoplasmic membrane.

### 3D modeling and mutational analyses of CcmB

**Figure 7:**
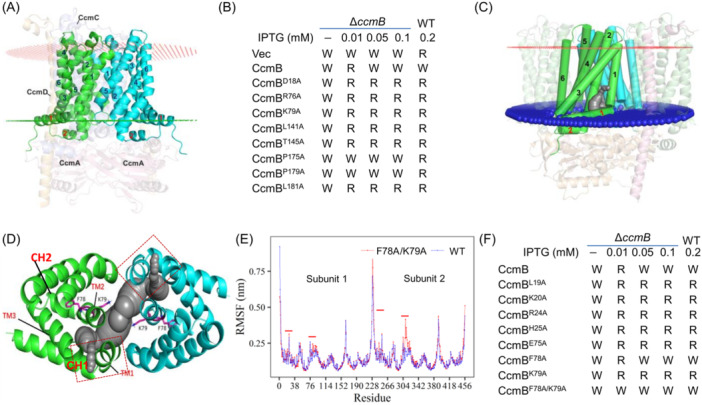
3D modeling and mutational analysis of CcmB. (A) The CcmB dimer in the Ccm(AB)_2_CD complex. The helix bundle is composed of 6 TM α‐helices (numbered in black) and 2 CH (cytoplasmic) α‐helices (numbered in red). CcmB dimer is in green and blue. The inner and outer leaflets of the membranes are shown in red lines and green dots, respectively. (B) Characterization of CcmB variants in terms of CCM. (C) A cavity (in gray) formed in the dimer interface of CcmB is predicted for heme. (D) The details of the cavity. The conjunction of CH1 and TM1 is likely the heme entrance from the membrane. Two residues, F78 and K79, extrude into the cavity. (E) RMSF analysis of CcmB and CcmB^F78A/K79A^ dimers. Two subunits are shown separately. Peaks correspond to the linker fragments connecting two neighboring helices. Peaks under red bars indicate the locations of the segments that are predicted to be important for heme entry. CcmB^F78A/K79A^ makes the subunit more flexible. (F) Characterization of CcmB variants in terms of the cyt *c* content. R, reddish; RMSF, root‐mean‐square fluctuation; W, whitish.

The structures of monomer CcmB, within the CcmABCD complex revealed by cryo‐EM (PDB ID: 7F02) and predicted with AF2, are highly similar, both of which mimic a six‐TM‐helix bundle, and during dimerization, two monomers assemble into a rigid 12‐helix bundle (Figures 7A and S6A,B). In addition, two cytoplasmic α‐helices, CH1 and CH2, are associated with the cytoplasmic side of the membrane in the direction parallel to the membrane (Figure 7A). Within each of these α‐helices, there are a number of perfectly or highly conserved residues in the CcmB homologs of representative bacteria hosting System I (Figure S6C). To investigate the mechanism underpinning the heme efflux mediated by CcmB, 28 out of these conserved residues were subjected to alanine scanning analysis (Figure S6C). The results revealed that all of the resultant CcmB variants were expressed similarly compared to CcmB^WT^ and a majority of them also behaved similarly (Figures S7A,B), indicating that CcmB is rather stable structurally. However, several retained CcmB activity for CCM but lost the ability to impair the process when overproduced, including D18 of CH1, R76, and K79 of TM2, L141, and T145 of TM4, and L181 of TM5 (Figures 7B and S7A). In addition, two variants, P175 and P179 of TM5, were even unable to complement the CcmB loss. As these residues are distributed throughout the entire protein, it would be reasonable to conclude that all of the helices are important to CcmB function.

In the CcmABCD complex, the helix‐bundle structure of CcmB is tightly packed^9^, ^10^, rendering the protein incapable of expelling heme. As a result, all probable conformations from published cryo‐EM structures are similar, lacking a pocket or a channel that allows heme to travel upwards from the cytoplasmic side of the membrane (Figures S7C,D). In contrast, a cavity was found in the AF2‐predicted dimer (Figure 7C). The cavity, located near the cytoplasmic side of the membrane, is surrounded by TM1, TM2, and TM3 as well as CH1 of each CcmB monomer. Apparently, a portion of TM2 extrudes into the cavity, residues F78 and K79 in particular (Figure 7D). The location of the cavity, similar to that of the heme pocket observed in heme transporter CydDC, is consistent with the intercalating behavior of heme to the lipid bilayers^36^, ^37^. Additionally, a potential entrance to the cavity was identified, which is a small triangle opening that is surrounded by CH1 (parallel to the membrane), TM1, and TM3 of each monomer and exposed to the hydrophobic interior of the membrane (Figure 7C,D).

To provide evidence to further support this observation, all‐atom molecular dynamics (MD) simulations were performed on the CcmB model, in which the structure of CcmB predicted with AF2 was used in the complex with heterogenous bilayers by CHARMM‐GUI. The analysis of the CcmB^WT^ dimer in the membrane (1000 ns, three times) using root‐mean‐square fluctuation (RMSF) revealed that the linkers (residues 48–51, 84–86, 160–165, 193–196) connecting any two helices had average values significantly higher than those of the helices (Figure 7E). Despite this, the first linker (residues 25–28) had a rather low average RMSF value (0.148 nm), suggesting that these residues are actually stable. In contrast, the last few residues (residues 21–24) of CH1 were rather flexible (average RMSF, 0.182; R24, in particular, has an average RMSF of 0.298) (Figure S7E). Similarly, we found that the last several residues of TM2 (residues 75–79, peak RMSF at F76, 0.260) were more flexible than the linker that follows (residues 83–97, 0.173) (Figure S7E). These data suggest that the flexible residues may play an important role in heme translocation from the membrane.

Impacts of residues of 21–28 and 75–84 on CcmB activity were then examined by alanine scanning analysis (Figures 7F and S6C). As shown in Figure 7F, all of these CcmB variants could function as CcmB for CCM. However, when the mutations that occurred in the CH1 and TM2, especially with the residues differing from alanine profoundly, they prevented CcmB from causing the detrimental effect. Surprisingly, when both F78 and K79 were replaced by alanine, the resultant CcmB variant (CcmB^F78A/K79A^) became more robust to expel heme as only residual leaky expression (IPTG‐free), which was barely detectable by western blot analysis, was able to fill in the CcmB loss (Figure S7F). To further assess the impact of the F78A/K79A double mutations on the structure, a model of the CcmB^F78A/K79A^ dimer within the membrane was constructed, and the dynamics changes induced by the mutations were evaluated by using RMSF, root‐mean‐square deviation (RMSD), dynamic cross‐correlation matrix (DCCM), and dictionary of secondary structure of protein (DSSP), which compared the behavior of the CcmB^F78A/K79A^ variant dimer with that of the CcmB^WT^ dimer within the membrane environment from different angles. The results revealed that the RMSD values of the WT and mutant CcmB were stable at about 0.3 nm after 200 ns and the average RMSD values were comparable, 0.293 and 0.303 nm for CcmB^WT^ and CcmB^F78A/K79A^, respectively (Figure S8A), suggesting that overall structure of CcmB is rather stable. However, the RMSF values of residues 75–95 were substantially different after double mutations (Figures 7E and S8B,C). The localized instability was also observed in dynamic secondary structure compositions revealed by DSSP. Compared to the WT counterparts, residues 74–77 in the monomer of CcmB^F78A/K79A^ were more inclined to form alpha‐helix and the secondary structure of residues 78–92 was less stable (Figure S9). Additionally, the DCCM analysis illustrated that the motion correlation between two regions of residues 75–108 and 156–172, located near the membrane interface, was weakened in CcmB^F78A/K79A^, either within a monomer or between monomers (Figure S10). While all of these data reinforce that the overall conformation of CcmB is stable, it is clear that F78A/K79A double mutations substantially increase the flexibility of the region around F78A/K79A and change the motion correlation of local regions.

**Figure 8:**
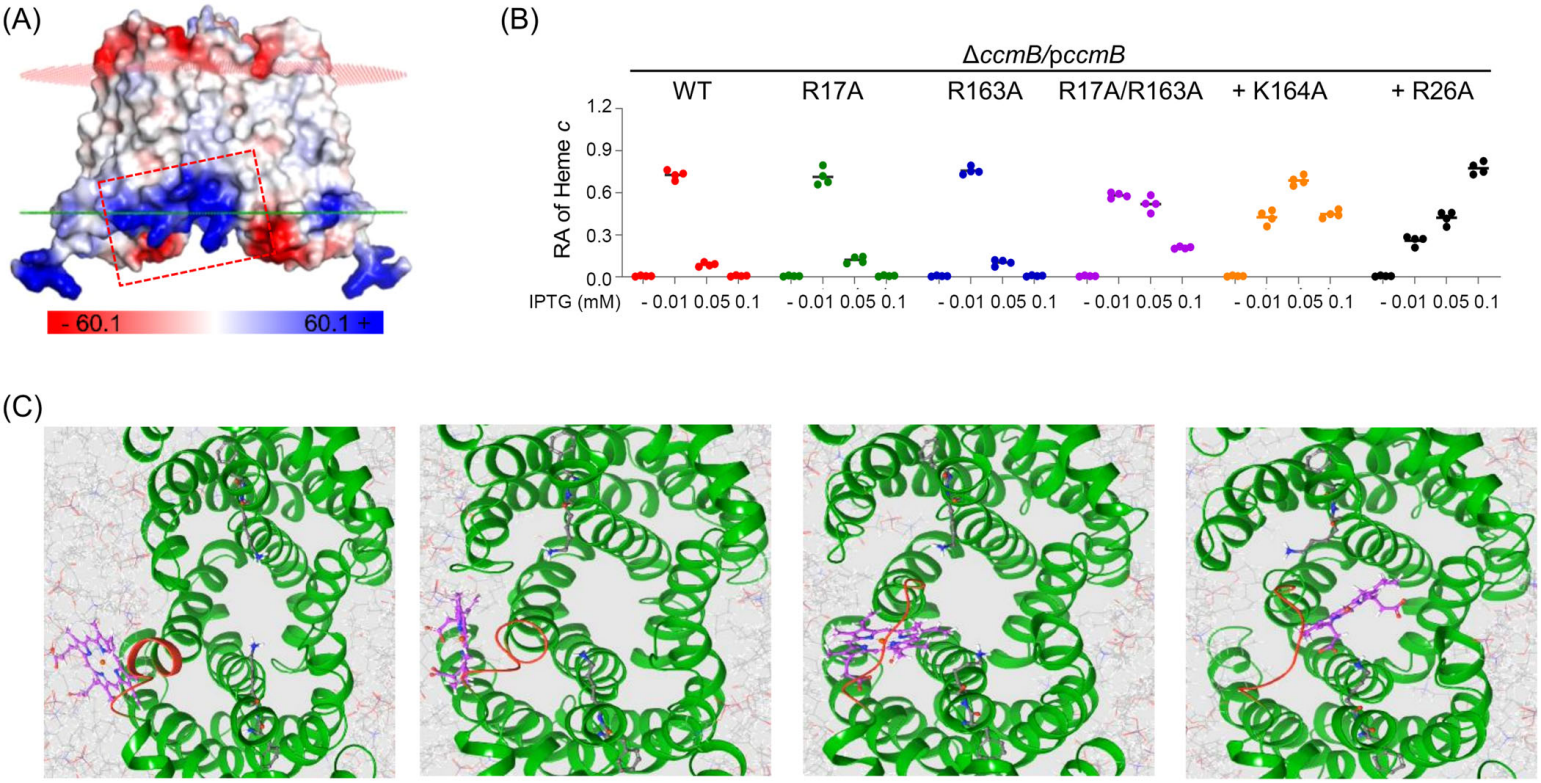
Structural features for heme entry in CcmB. (A) The vacuum electrostatics of fractional CcmB and the protein surface visualized by assigning colors based on their charge distribution. The predicted heme entry is boxed. (B) The cyt *c* content of Δ*ccmB* expressing one of the CcmB variants. +K164A and +R26A represent R17A/R163A/K164A and R17A/R163A/K164A/R26A variants, respectively. (C) Close‐up views showing that heme interacts with CcmB to enter the binding cavity. The drastic changes in CH1 are shown in red. These snapshots were taken from MD simulations.

To explore how heme enters the cavity of the CcmB dimer, we carried out a structural comparison between CcmB and CydDC. In the case of CydDC, the porphyrin scaffold of heme tends to enter with the hydrophobic TM‐helix buddle, and the two hydrophilic propionate groups stick out the membrane into the cytoplasm to interact with the residues near the membrane surface (Figure S11A). To facilitate heme absorption, many of the surface residues of CydDC are positively charged (Figure S11B). A similar distribution of positively charged residues on the surface was observed in CcmB, including R17, K20, R24, H25, R26, R163, and K164 within the marked region (Figure 8A). Among them, K20, R24, and H25 have been shown to be critical for heme efflux (Figure 7F). Although the impacts of the rest of these residues on the cyt *c* content of ∆*ccmB* were rather minor, their effects were additive (Figure 8B), supporting their involvement in heme absorption. Furthermore, the 3D heme interacting model for CcmB was constructed computationally through a template‐based approach and MD simulations with CcmB structures were reported or predicted with AF2. *C. diphtheriae* HrtAB and CydDC were used as the templates because they support heme translocation through the membrane^34^, ^37^. From the free energy profile after reweighting obtained with the metadynamics, a model was generated to show that heme could enter the cavity autonomously and during heme moving conformational changes in the CH1 helix, especially around residues 21–25, are constant and substantial (Figure 8C) (Supporting Information: movie 1). In addition, by using AutoDock Vina, we further refined MD simulations and found that the heme molecule entered the pocket, and remained centered and confined within the pocket stably (Supporting Information: movie 2). All of these data support that CcmB has a pocket for heme, which is primarily, if not exclusively, used to draw heme from the membrane. In addition, the similarity in the location of the heme pocket and the distribution of positively charged residues between CydDC and CcmB suggests that these two transporters extract heme from the membrane through a similar mechanism.

## DISCUSSION

In bacteria, heme is synthesized in the cytoplasm and has to be translocated across the cytoplasmic membrane to the CCM apparatus through a transmembrane transporter^38^. Two hypotheses for heme transport in System I were proposed two decades ago based on available data then. One suggests that CcmC, which is not a subunit of the CcmAB transporter, solely is responsible for translocating heme to CcmE through an unknown pathway^11^, ^39^. However, in contrast to CcsBA of System II, CcmC lacks a specific heme channel^12^, ^29^. The other is that heme is exported through the CcmABCD complex, presumably by the CcmAB core transporter, to the periplasmic WxWD domain of CcmC, which in turn delivers heme to CcmE^38^. Two decades later, despite the recent advances in structure illustrations of the CcmABCD complex^9^, ^10^, we still know little about how heme is translocated for CCM.

In this study, using *S. oneidensis* as a suitable research model and a high‐throughput approach to screen for proteins regulating the cyt *c* content, we identified CcmB in excess to inhibit CCM. Relatively comprehensive genetic, biochemical, and computational analyses uncovered that CcmB alone acts as a heme translocase, but when in complexation with CcmACD translocates heme from the cytoplasmic membrane to CcmC exclusively, filling in the missing link in our understanding of the heme translocation for CCM.

Heme‐dedicated efflux pumps in the cytoplasmic membrane have been identified and characterized extensively in Gram‐positive pathogens, especially *Staphyloccus aureus* and *C. diphtheriae* ^33^, ^40^, ^41^. It is proposed that these bacteria have evolved the heme efflux machinery because their cytoplasmic membrane, the primary target of heme toxicity, may be exposed to high concentrations of heme from lysed erythrocytes^31^, ^32^, ^33^, ^34^. In contrast, Gram‐negative bacteria lack a homolog of this system. This can be readily explained by that the OM is not permeable to heme, and importing heme as an iron source requires a specific TonB‐dependent transporter to translocate heme across the OM to reach the IM and is tightly regulated^42^. Our data indicate that CcmB alone is a highly effective heme‐dedicated translocase in Gram‐negative bacteria hosting System I. If unchecked, this activity appears to be extremely detrimental to the general physiology by causing severely lowered intracellular heme content. Although the impact is clearly pleiotropic given that many biological processes entail hemoproteins, we believe that the growth defect caused by excess CcmB is largely due to the reduced activity of the cyt *bd* oxidase. In *S. oneidensis* cells grown under normal conditions, the cyt *cbb*_3_ oxidase dictates oxygen respiration and the cyt *bd* oxidase plays an auxiliary role^22^, ^24^, ^43^, ^44^. Our data presented here show that the modest overproduction of CcmB abolishes CCM, resulting in the loss of the cyt *cbb*_3_ oxidase, but further overproduction is required to impair the cyt *bd* oxidase.

The unnoticeable heme efflux in bacteria hosting System I convinces us that evolution has honed CcmB and the ABCD complex for their role in CCM. First, CcmB possessing heme‐exporting activity is always encoded by the second gene of the *ccmABCD* operon (or the *ccmABCDE* operon in most cases), ensuring that CcmB is produced for a correct stoichiometry. One may imagine that this arrangement may also have an impact on the correct and swift formation of the CcmABCD complex, which merits further investigation. Second, although the configuration of the ABCD complex, either Ccm(AB)_2_CD or Ccm(ABCD)_2_, is not yet certain^9^, ^10^, CcmAB is the core of this complex, to which CcmCD is bound tightly. As a result, the required conformational changes of CcmB that expel heme from the membrane, which are key to the heme efflux activity of HrtBA and CydDC, are not allowed^34^, ^37^. Third, the complexation ensures CcmC and CcmB are close enough so that heme can be only directed toward the heme‐handling domain (WxWD) domain of CcmC. This idea gains support from the finding that CcmB enhances heme efflux when the heme‐holding capacity of CcmC is compromised. Finally, the detectable impact of CcmD on CcmB‐mediated heme efflux may be a result of stabilizing the CcmABCD complex.

The primary, if not exclusive, heme source of CcmB turns out to be the IM, coinciding with the findings of HrtBA and CydDC^34^, ^37^. HrtB, the permease subunit, adopts a four‐helix TM bundle structure and does not directly receive heme from the cytoplasm because the bundle lacks a substrate‐translocating cavity across the membrane^34^. Instead, the TM bundle monomers contact each other to generate a lateral access in the vicinity of the lateral membrane outer leaflet for heme association. While the location of the heme‐binding site in the TM helices of both CydDC and CcmB is close to the membrane inner leaflet, it follows the same law controlling the intercalation of heme in the lipid layer^37^. On one hand, the porphyrin scaffold of heme is lipophilic and tends to be embedded into the middle of the membrane, establishing interactions with the acyl chains of the lipids. On the other hand, the two propionate groups of heme are oriented toward the lipid head groups, predominantly interacting with the ethanolamine groups of phosphatidylethanolamine molecules. To promote heme absorption, both CydDC and CcmB expose many positively charged residues on the surface around the heme pocket to enhance interaction with heme. In addition, conformational changes in certain helices of both CydDC and CcmB revealed by MD analysis are required for heme entry, TM4 of the former, and CH1 of the latter. These data conclude that CcmB takes heme from the membrane via a trap‐and‐flip mechanism similar to that described for CydDC and other active lipid transporters^37^, ^45^, ^46^.

The uptake of heme by HrtB or CydDC from the membrane is not dependent on ATP hydrolysis, supporting that at least the substrate binding and occlusion is an autonomous process^34^, ^37^. In fact, even the subsequent exporting process does not require the energy released from ATP hydrolysis because our data demonstrated that HrtB alone, similar to CcmB, can expel heme from the membrane. This may offer an explanation for the enigma that overproduced CcmC is capable of attaching heme covalently to CcmE^11^. CcmC per se may be able to extract heme from the membrane independent of ATP hydrolysis. As this activity is conceivably rather weak and heme would be immediately held in the WxWD heme‐binding domain, no heme would be exported into the periplasm. More importantly, heme translocation across the membrane by CcsBA of System II does not require an energy source either^12^. Based on all of these recent findings, we envision that the electrochemical potential likely provides a source of energy to drive the heme efflux by CcmB^47^, or more radically that energy may not be required for CcmB‐mediated heme translocation.

The membrane localization of heme is demonstrated to be an autonomous reaction, largely owing to the hydrophobicity of the molecule^48^. Our recent work revealed that CcmB interacts with heme‐trafficking protein HtpA directly, and based on this, we have proposed that HtpA promotes CCM by supplying heme to the CcmABCD complex^21^. However, the BioID screening revealed that the majority of the CCM components would be in proximity to HtpA. By combining the data in this study, it appears more likely that HtpA delivers heme to the membrane in the vicinity of the CcmABCD complex, resulting in an increased abundance of heme in the region, which in turn enhances heme supply to CCM.

## MATERIALS AND METHODS

### Bacterial strains, plasmids, and culture conditions

The bacterial strains and plasmids used in this study are listed in Table S2. The sequences of the primers used in this study are available upon request. All chemicals were purchased commercially, mainly from Sigma‐Aldrich unless otherwise noted. For genetic manipulation, *E. coli* and *S. oneidensis* were grown in Lennox broth (LB; Difco) under aerobic conditions at 37°C and 30°C, respectively. When needed, 2,6‐daminopimelic acid (DAP), ampicillin (Amp), kanamycin (Km), and gentamicin (Gm), were supplemented at 0.3 mM, 100 μg/ml, 50 μg/ml, and 15 μg/ml, respectively.

For aerobic growth, both LB and defined medium MS containing 30 mM lactate as the electron donor were used but similar results were obtained^49^, and the data from LB were presented unless otherwise noted. For growth measurement, fresh medium was inoculated with cultures grown to the late‐exponential phase (OD_600_ = 0.6) to an OD_600_ of ∼0.01 and shaken at 200 rpm at 30°C.

### Mutant construction and complementation

Construction of in‐frame deletion mutants for *S. oneidensis* was performed with the *att*‐based Fusion PCR method essentially the same as described previously^50^. In brief, a mutation construct was generated by two rounds of PCR, which was introduced into plasmid pHGM01, and the resulting vectors were maintained in *E. coli* DAP auxotroph WM3064. After subsequently being transferred into relevant *S. oneidensis* strains via conjugation, integration of the deletion constructs into the chromosome was selected by resistance to gentamicin and confirmed by PCR. Correct transconjugants were then subjected to sucrose counterselection and gentamicin‐sensitive and sucrose‐resistant colonies were screened by PCR for the intended deletions and verified by sequencing.

Genetic complementation of the mutants was carried out with pHGEN‐P*tac* mostly, in which the gene of interest was placed after IPTG inducible promoter P_*tac*_ ^51^. In addition, the P_*arcA*_, which is modestly and constitutively active under growing condition used in this study, was used^52^, ^53^. The resultant vectors verified by sequencing were transferred into the relevant strains via conjugation.

#### Site‐directed mutagenesis

Genes of interest were cloned into pHGEN‐P*tac* using the standard protocols. Site‐directed mutagenesis was performed with Quick‐Change Kit (Agilent) according to the manufacturer's guidelines. All substitutions were verified by DNA sequencing.

### The whole genome library construction and gene screening

*S. oneidensis* genomic DNA was partially digested with *Sau*3AI, separated by agarose gel electrophoresis, and DNA fragments of 0.8‐4 kb were recovered from the gels and ligated to *Bam*HI digested pHGEN‐P*tac*. Ligated plasmid DNA was introduced into *S. oneidensis* WT by electroporation^54^. The transformants were plated onto Gm‐containing LB agar plates supplemented with IPTG at 0.1 or 0.5 mM. From ∼30,000 colonies grown with 0.1 mM IPTG, we obtained two colonies that harbor potential genes. Plasmids from these two isolates were extracted and the insertion sequences were determined.

#### Confocal microscopy and fluorescence measurement

To determine the cellular location of overproduced CcmB, DNA fragments encoding CcmB‐GFP fusion proteins were cloned into pHGEN‐P*tac* and transferred to the *S. oneidensis* WT strain. Cells expressing the GFP fusion proteins were grown to the mid‐exponential phase (OD_600_ = 0.4) and visualized with LSM710nlo laser confocal microscope (Carl Zeiss) with verified cytoplasmic FlhG and inner‐membrane protein PetA of *S. oneidensis* as the control^55^, ^56^.

#### Heme quantification assay

Cultures of *S. oneidensis* strains grown in LB to the early stationary phase were aliquoted to contain similar numbers of cells and centrifuged. The resulting pellets were photographed and subsequently subjected to heme quantification. Total heme and heme *c* were quantified essentially the same as described before^21^. Both the QuantiChrom heme assay kit (BioAssay Systems) and the Turbo‐TMB (3,3′,5,5′‐tetramethylbenzidine) assay were used in this study^26^. Protein concentration of the cell lysates was rapidly assessed by using a GE NanoVue Spectrophotometer and/or determined using a Bradford assay (Bio‐Rad) according to the manufacturer's instructions. The standard curve was generated with heme solutions from 4 to 150 nM. For heme *c* measurement, the proteome of the culture was extracted and denatured by trichloroacetic acid (TCA) (8%, final concentration) precipitation to release non‐covalently attached heme, and the precipitated protein portion was assayed. The intracellular heme content was derived from the difference in concentrations of total heme and heme *c*. Extracellular heme levels were directly determined with the QuantiChrom heme assay kit.

### Construction of heme biosensor

A heme biosensor, which utilizes *Synechocystis* HO and smURFP, was constructed according to the design described before^57^. DNA sequences for both proteins were synthesized and introduced into expression vector pHGEN‐P*tac*. While the HO gene was placed after IPTG‐inducible promoter P_*tac*_, constitutive active promoter P_*arcA*_ was used to drive the expression of the smURFP gene^51^, ^52^. For control experiments, each of these genes was cloned into the same vector alone under the same design. Strains carrying the vector grown to late‐exponential phase (OD_600_ = 0.8) were induced to produce HO by the addition of 0.2 mM IPTG, and cells were collected 20 min later for fluorescence measurement, which was performed on TECAN infinite M200PRO (excitation/emission, 642/670 nm)^27^.

#### Expression assay

Expression of the gene of interest was assessed by assaying the activity of its promoter using a single‐copy integrative *lacZ* reporter system, as described previously^22^. Constructs for the assay were from previous studies^19^, ^21^, ^24^.

#### Nitrite susceptibility assay

Spotting assay was applied to assay nitrite sensitivity, which reflects cyt *bd* activity, on LB plates as described before^22^, ^58^. Cultures at the mid‐exponent phase were adjusted to approximately 10^8^ CFU/ml (dilution factor, 0), followed by 10‐fold serial dilution. A 5 μl of each dilution was spotted onto plates, which were incubated at 30°C for at least 24 h before being photographed.

#### Hydrogen peroxide susceptibility assay

H_2_O_2_ consumption assay was employed to assess the catalase activity as before^23^, ^59^. The concentrations of H_2_O_2_ were determined by FOX‐assay method using TECAN infinite M200PRO^60^. Susceptibility of *S. oneidensis* strains to H_2_O_2_ was assayed by growing cells in a 24‐well plate containing H_2_O_2_ at various concentrations.

#### SDS‐PAGE and western blot analysis

Cells were prepared the same as for heme quantification, lysed by sonication with a Xinzhi Sonifier (JY92‐IIDN; NingBo) at maximum output on ice until no whole bacterial cells were visible, and the protein extracts were collected after centrifugation at 3000*g* for 10 min. Envelope fractions were prepared essentially the same as previously described^61^. Protein extracts were resolved by 12% SDS‐PAGE, and subsequently either stained with Coomassie Brilliant Blue R‐250 or transferred onto the PVDF membrane for western blot analysis. The blotting membrane was probed with mouse anti‐His_6_‐tag or anti‐GFP and goat anti‐mouse IgG‐HRP (1:100,000) as the primary and secondary antibodies (Beyotime), respectively. The blots were developed with a SuperSignal West Dura Extended Duration Substrate kit (Invitrogen) and visualized with a ChemiScope 6000 chemiluminescence imaging system (Clinx).

### In silico analysis and statistics

CcmB homologs with different BLASTp *E*‐values as cutoffs were identified by BLASTp and HMMER^62^. Sequence alignments were generated with ClusterIX^62^. The predicted structure of CcmB was obtained from AlphaFold Protein Structure Database or predicted by AF2 locally^63^. Distribution of the homologs and similarity comparison of CcmB homologs were analyzed with HMMER. Sequence similarity networks (SSNs) for the CcmB homologs were generated by the EFI‐Enzyme similarity Tool on the EFI site (https://efi.igb.illinois.edu)^30^. To refine the sequences, proteins over 300 amino acids were removed and those from bacteria that have neighborhood regions identical to CcmABCD (predicted by EFI‐Genome Neighborhood Tool, EFI‐GNT) were added by a sequence identity cutoff threshold of 0.95 using Jalview. The resulting list was applied to the EFI‐Enzyme Similarity Tool (EFI‐EST) for the network analysis and figures were generated using Cytoscape 3.9.1.

For MD simulations, heterogenous bilayers consisting of 1‐palmitoyl‐2‐oleoyl‐*sn*‐glycero‐phosphoethanolamine (POPE, 75%), 1‐palmitoyl‐2‐oleoyl‐*sn*‐glycero‐3‐phosphoglycerol (POPG, 20%), and 1‐palmitoyl‐2‐oleoyl‐cardiolipin (POCL1, 5%) were generated and the resulting bilayers contained a planar region with a water thickness of 2.25 nm along the *Z*‐axis on top and bottom of a rectangular box and were equilibrated with 150 mM KCl electrolyte to pH 7.0^64^, ^65^. The AF2‐predicted CcmB^WT^ dimer was introduced into the bilayers by CHARMM‐GUI. All‐atom CHARMM36 force field was used for lipids, ions, protein, and TIP3P water and all unbiased simulations were performed using GROMACS‐v2023^66^. The system was energetically minimized with the 2000 steepest descent steps and equilibrated gradually in the NPT ensemble at a temperature of 310 K using mdp files from CHARMM‐GUI (generated grid information for PME FFT automatically). The structure files of the protein‐lipid system were saved for further analysis. A production run was performed in the NPT ensemble at a temperature of 310 K and a pressure of 1 bar for a total of 1000 ns and repeated three times. Couplings of temperature and semi‐isotropic pressure were carried out by using the velocity‐rescale method (time constant of 1 ps) and the Parrinello‐Rahman algorithm (time constant of 5 ps), respectively. The model building and MD process of CcmB^F78A/K79A^ are the same as CcmB^WT^, whose dimer was mutated by CHARMM‐GUI based on the CcmB^WT^ structure. The ccmB^WT^ dimer used for docking was extracted from the MD simulation trajectory described above. The ideal heme structure was obtained from the Protein Data Bank (PDB). The ligand‐protein docking was performed using AutoDock Vina software^67^. The highest scoring docked conformation was then introduced into heterogenous bilayers using the CHARMM‐GUI by the approach described recently^37^. The CHARMM36m force field was employed, and the processes included minimization, equilibration, and production, following the same protocols as described above. All‐atom MD simulation for 100 ns was conducted to monitor dynamic changes in the conformation of the system.

To explore potential heme entrance, the final frame of the 100 ns MD simulation was extracted and used as the initial conformation for a 700 ns well‐tempered metadynamics simulation in Desmond^68^. The distance between the pocket center and the iron atom of the heme was defined as the collective variables. The parameters for the metadynamics simulation, including width, height, energy, and interval, were set to 0.05, 0.03, 1.2, and 0.09, respectively, and the ensemble was set to NPT (300 K temperature and 1 bar pressure). MD simulation trajectories were analyzed. RMSD and RMSF of backbone were analyzed by GROMACS‐v2023, and DCCM and DSSP were analyzed using Bio3D and VMD‐v1.9.3, respectively^69^, ^70^. Additionally, the images of structures were operated on PyMOL^71^.

Statistical significance was calculated by using two‐tailed Student's *t*‐test for pairwise comparisons and using ordinary one‐way analysis of variance with the Student–Newman–Keuls multiple comparisons and Tukey post hoc test for determining significance between all groups using GraphPad Prism 9.3.1 (GraphPad). Data values are presented as mean ± standard deviation (SD).

## AUTHOR CONTRIBUTIONS

**Yuanyou Xu**: Formal analysis (equal); investigation (equal); software (equal); validation (equal); writing—original draft (equal); writing—review and editing (equal). **Wei Wang**: Formal analysis (equal); investigation (equal); validation (equal); writing—original draft (equal). **Qianrou Zhang**: Investigation (equal). **Sirui Han**: Investigation (equal). **Jiahao Wang**: Investigation (supporting). **Shihua Wu**: Funding acquisition (supporting); project administration (supporting); resources (supporting); writing—original draft (supporting); writing—review and editing (supporting). **Haichun Gao**: Conceptualization (lead); funding acquisition (lead); project administration (lead); resources (lead); writing—review and editing (lead).

## ETHICS STATEMENT

No animal or human research was involved in this study.

## CONFLICT OF INTERESTS

The authors declare no conflict of interests.

## Supporting information

Supporting information.

Supporting information.

Supporting information.

## Data Availability

The raw data for MD have been deposited and are available at https://doi.org/10.5281/zenodo.11631464. All other data supporting the findings of this study are available within the article and its supporting information materials.
