# Supplementary material for: Complexation of CcmB with CcmACD safeguards heme translocation for cytochrome c maturation
Source: mLife. 2025 Jan 6;4(1):29–44. doi: 10.1002/mlf2.12150 (PMC11868835; doi:10.1002/mlf2.12150)
Supplement: Supplementary file 3 — Supporting information. [file MLF2-4-29-s002.pdf]

微生物 mLife Open Access

Supporting Information for

Complexation of CcmB with CcmACD safeguards heme translocation for cytochrome *c* maturation

Yuanyou Xu,[1] Wei Wang,[1] Qianrou Zhang, Sirui Han, Jiahao Wang, Shihua Wu[2] and Haichun Gao,[2]

Institute of Microbiology and College of Life Sciences, Zhejiang University, Hangzhou, Zhejiang, 310058, China

[1]Y.X. and W.W. contributed equally to this work.

[2]To whom correspondence may be addressed.

Shihua Wu

drwushihua@zju.edu.cn

Haichun Gao

haichung@zju.edu.cn

This PDF file includes:

- Supporting text
- Figures S1 to S11
- Tables S1 to S2

Other supporting materials for this manuscript include the following:

- Movies S1 to S2

Supplementary Text

Heme-efflux activity of CcmB is highly conserved in bacteria hosting System I

In order to determine whether the heme-efflux activity is a common feature of CcmB homologs, we analyzed CcmB proteins with multiple bioinformatics programs, including BLASTp, HMMER, EFI, and AlphaFold2 (AF2)[1-3]. With the cutoff set as a BLASTp E-value of 1e-10, HMMER analysis returned 2380 homologous proteins from organisms within all three domains and even viruses (Fig. S4A). Despite this, only 10, 6, and 1 are from Archaea, Eukaryota, and Viruses respectively, and most of them have a E-value (< 1e-25) and/or differ from *S. oneidensis* CcmB substantially in protein length (Fig. S4A; Table S1). Among these homologs, 2242 belonged to proteobacteria, including 1156, 839, and 227 from α-, γ-, and β-proteobacteria respectively (Fig. S4b), a result that matches well with the distribution of System I, suggesting that the *ccmB* genes co-evolve with other *ccm* genes mostly. Notably, the CcmB homolog (LOC109505412) of *Elaeis guineensis* (oil palm) is similar in length and has a BLASTp E-value of 3.5e-57, and a protein encoded by the next gene (LOC109505411) is highly homologous to CcmC (E-value: 2e-51). Moreover, these two proteins display extremely high level homology to CcmB and CcmC of *Hydrogenophaga* Sp. RAC07 (< 1e-110) respectively, a β-proteobacterium hosting System I (Table S1). More importantly, both genes included in the *E. guineensis* genome are intron-free and could not be assembled into 16 chromosomes or the plastid chromosome, implying a possibility of contamination during sequencing.

These CcmB homologs were further analyzed by constructing a sequence similarity network (SSN)[3]. A main cluster (cluster I) of nodes in this network contains most of CcmB homologs from bacteria hosting System I (Fig. 5A, 5B). The CcmB homologs in cluster are found in two closely connected subgroups, represented by α- (green dots around *R. capsulatus*) and β/γ-proteobacteria (purple and red/blue dots respectively). The SSN network also clearly showed that the CcmB homologs in the organisms beyond α-, γ-, and β-proteobacteria are grouped into distinct clusters in a rather diverse way, suggesting weak evolution linkages among them. Among these low-similarity CcmB homologs, it is conceivable that only a portion of them may be involved in CCM because prokaryotes are generally equipped with a large number of ABC transporters, of which the permease components share considerable sequence similarities[4]. We therefore focused on CcmB

homologs from the organisms hosting System I, mostly Archaea, as they form a distinct cluster (Cluster II). The archaeal CCB systems, as in *Methanosarcina acetivorans*, differ from the bacterial one in that they generally lack CcmD and CcmH as well as have a CcmE with a distinct motif (CXXXY instead of the typical CXXCH) for covalent attachment of heme[5-6]. However, none of the archeal CcmB homologs under test was able to complement the CcmB loss or to compromise CCM of the WT (Fig. 5C; Fig. S4C,S4D; Table S1). Given that the proteins were expressed (Fig. S4E), the result suggests that these CcmB homologs could not function as *S. oneidensis* CcmB to efflux heme, at least in *S. oneidensis* cells. In addition, we tested a few CcmB homologs from the organisms in other clusters, including *Dictyobacter kobayashii*, *Deinococcus radiodurans*, *Heava brasiliensis*, and Wolbachia phage WO. As expected, none of them was able to function as *S. oneidensis* CcmB (Fig. S4C-E; Table S1).

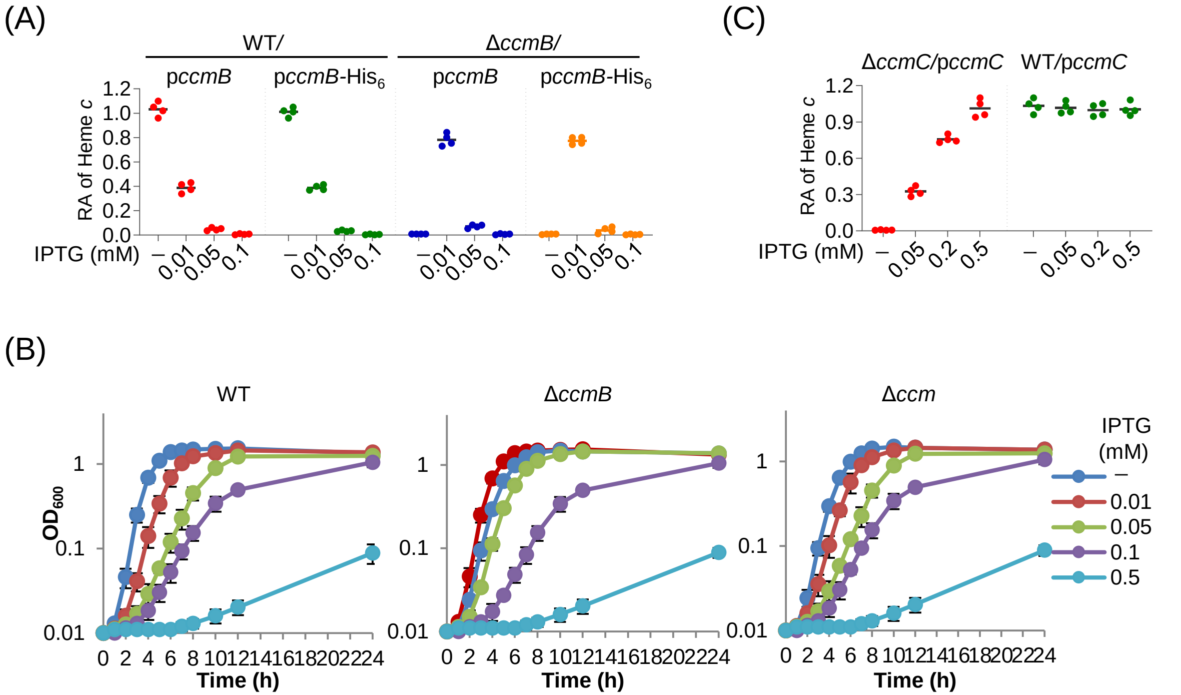

Figure S1. Physiological impacts of overexpressed CcmB or CcmC. (A) Effect of CcmB and CcmB-His_6 overexpression on the cyt *c* content. The early stationary phase cultures grown with IPTG at varying levels were collected for the assay as described in (Figure 2B). (B) Growth of indicated strains in LB under aerobic conditions. All strains carried a vector expressing *ccmB* driven by IPTG-inducible promoter P*tac*. Growth was represented by the optical density of the cultures at 600 nm (OD_{600}), experiments were performed four times, and the data were presented as the mean ± SD (error bar). (C) Effect of CcmC overexpression on the cyt *c* content.

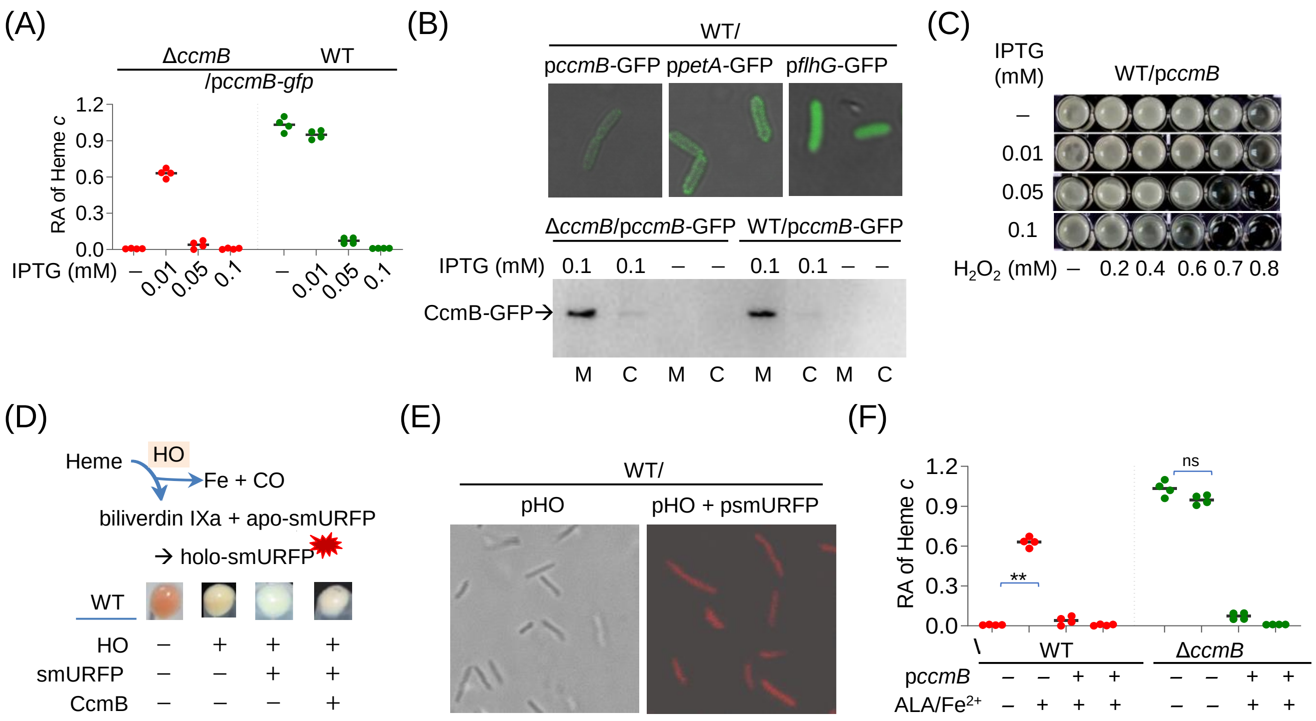

Figure S2. Localization and impacts of overexpressed CcmB. GFP was fused to the C-terminus of CcmB with a five-amino-acid linker (SGGSG), which ensures GFP folding without affecting CcmB configuration. (A) Impacts of CcmB-GFP on the cyt *c* content of relevant strains. (B) Localization of overexpressed CcmB. Upper: WT cells expressing CcmB-GFP were grown to the mid-exponential phase with 0.1 mM IPTG, and visualized with a confocal microscope. FlhG and PetA, verified cytoplasmic and inner-membrane proteins respectively, were used as the control. Lower: Cells were processed and subjected to Western blotting with antibodies against GFP. M, the membrane portion; C, the cytoplasm portion. (C) H_2O_2 sensitivity assay. Cells were grown in 1 ml LB supplemented with H_2O_2 up to 0.8 mM in a 24-well plate at 30 ºC. Shown were the results after 16 h of inoculation. All experiments were performed at least 3 times independently, and representative results were shown. (D) Heme biosensor. *Synechocystis* HO degrades heme to produce Biliverdin IXa, which activates smURFP. In WT, 0.2 mM IPTG was used for expression of indicated proteins. (E) *Synechocystis* HO and smURFP expressed *in vivo*. Representative image of smURFP expressed in WT. The observation was carried out under a confocal microscope (excitation/emission = 642/670 nm). (F) Intracellular heme levels in WT and Δ*ccmB* grown with the addition of ALA and Fe^{2+}. ITPG at 0.1 mM was used to induce *ccmB* expression. In all panels requiring statistics analysis, for the values compared, ns, not significant; *, $p < 0.05$; **, $p < 0.01$; ***, $p < 0.001$.

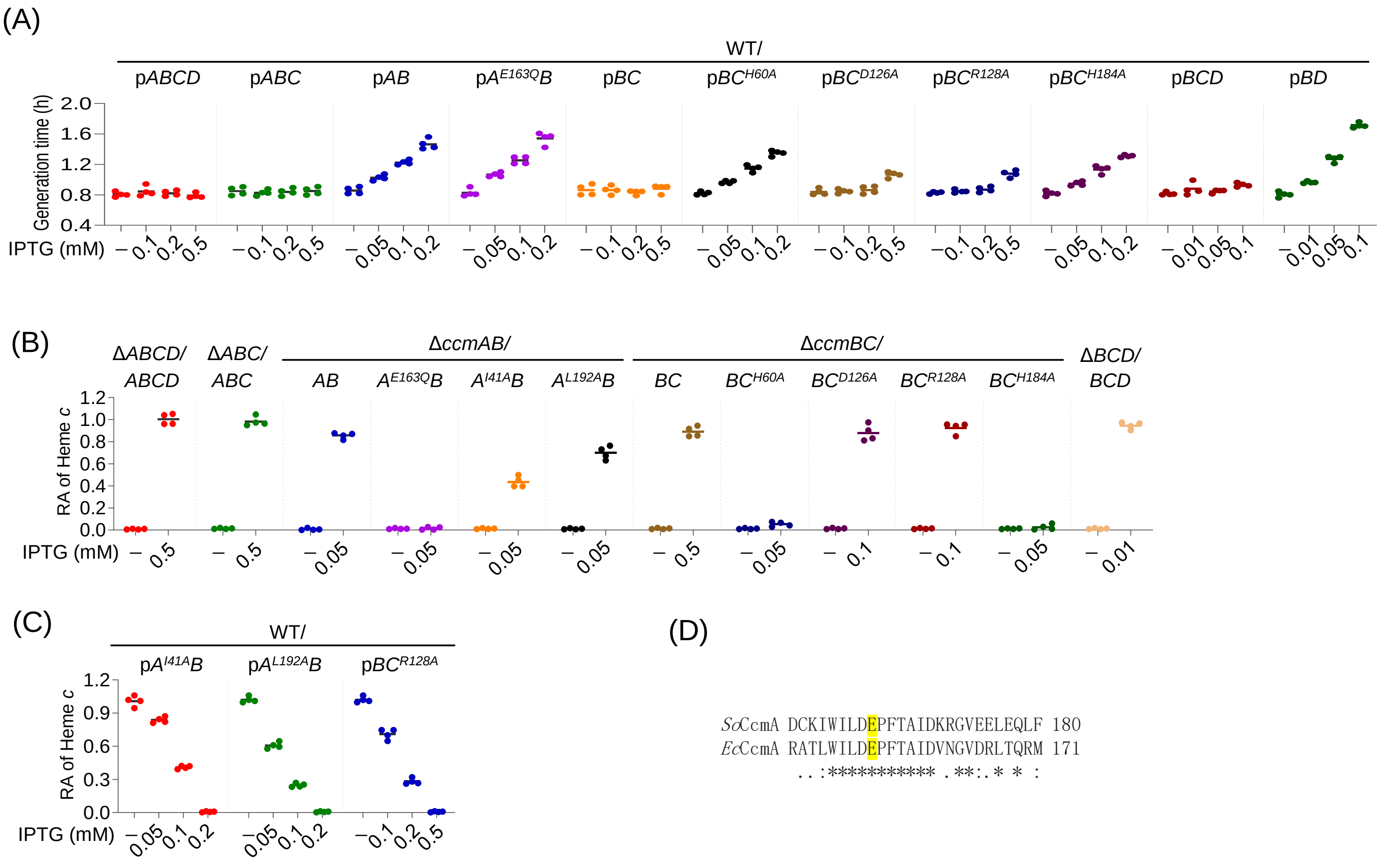

Figure S3. Complexation of CcmB with CcmACD safeguards heme translocated by CcmB to be used for CCM only. (A) Generation times of WT expressing *ccmABCD* genes in varying combination, including three CcmA and CcmC variants. (B) The cyt *c* content of indicated mutants expressing *ccmABCD* genes in varying combination. (C) The cyt *c* contents in WT expressing some CcmA and CcmC variants. (D) Sequence alignment of *S. oneidensis* and *E. coli* CcmA segments. Conserved E residues, which are essential to CcmA activity, are highlighted.

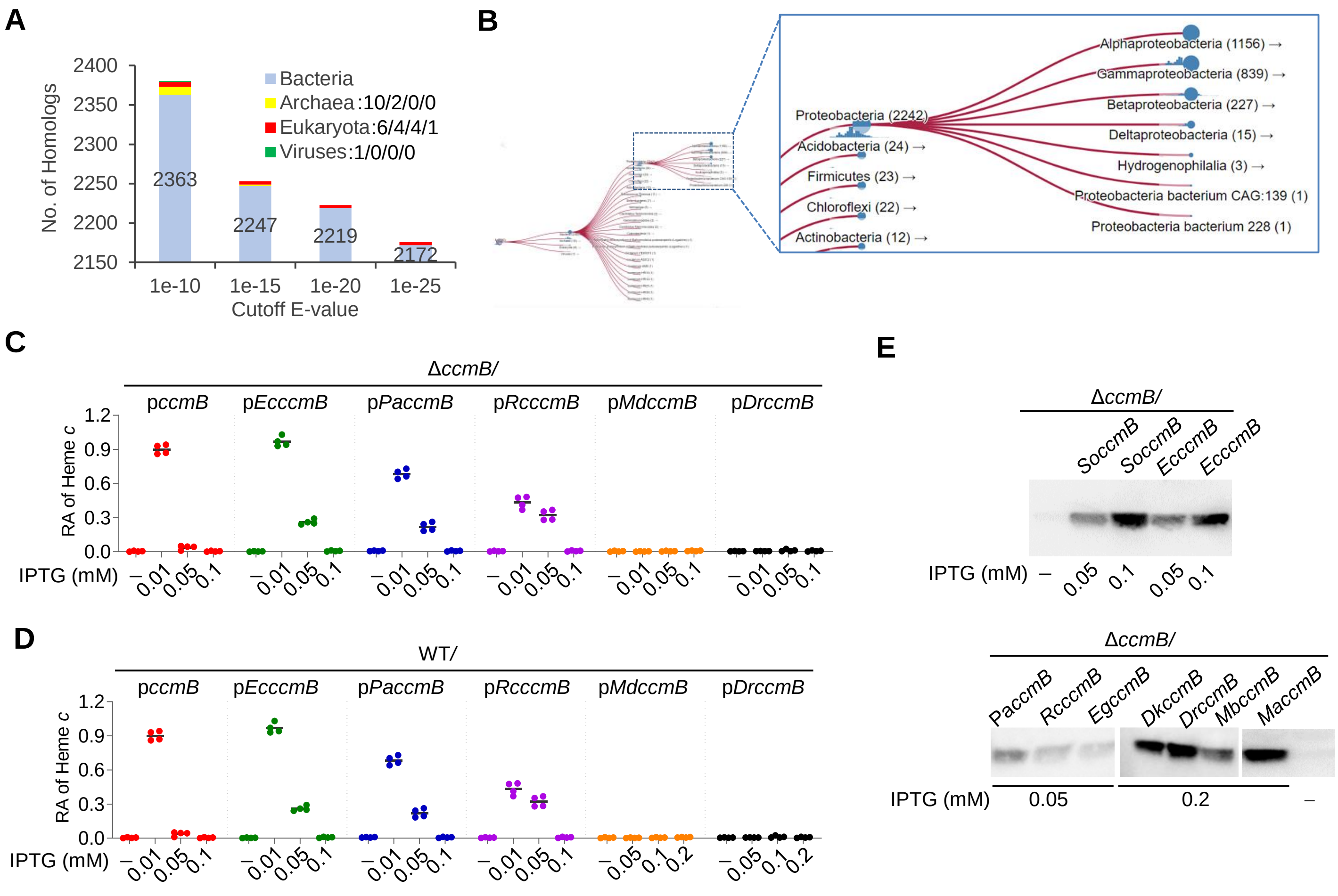

Figure S4. Physiological impacts of CcmB homologs on the cyt *c* content. (A) Cross-domain distribution of CcmB homologs. The numbers of CcmB homologs in each domain and viruses with different BLASTp E-values as the cutoffs are given. The analysis was carried out with HMMER. (B) The HMMER result with BLASTp E-value of 1e-10 as the cutoff. To show the numbers of species in proteobacteria, the relevant area is enlarged. (C) The cyt *c* content of Δ*ccmB* expressing one of CcmB homologs. (D) The cyt *c* content of WT expressing one of CcmB homologs. In both (C and D), cells were prepared and assayed the same as described (Figure 2B). (E) Expression of representative CcmB homologs assessed by Western blot. Upper panel, expression of CcmB of *S. oneidensis and E. coli*. Lower panel, CcmB from representative bacteria and archaea. *Pa*, *P. aeruginosa*; *Rc*, *R. capsulatus*; *Eg*, *E. guineensis*; *Dk*, *D. kobayashii*; *Dr*, *D. radiodurans*; *Mb*, *M. burtonii*; *Ma*, *M. acetivorans*. Refer to Table S1 for more information.

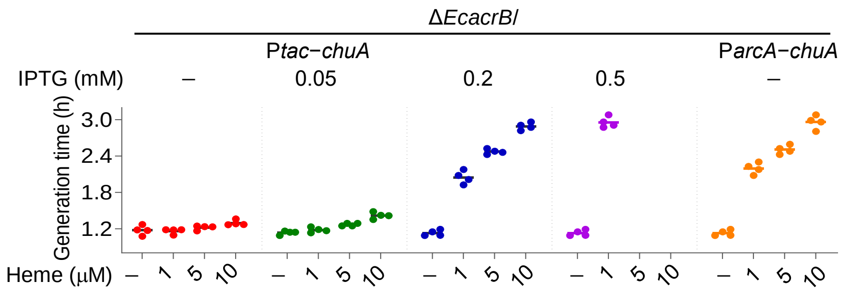

Figure S5. CcmB exports heme primarily from the cytoplasmic membrane. Growth of heme-sensitive *E. coli*. Heme-resistant Δ*EcacrB* can be sensitized to heme by expression of *chuA.* To find a proper promoter to drive expression, we determined the IPTG concentrations suitable for the study, and then chose a moderate constitutive promoter for the *S. oneidensis arcA* gene.

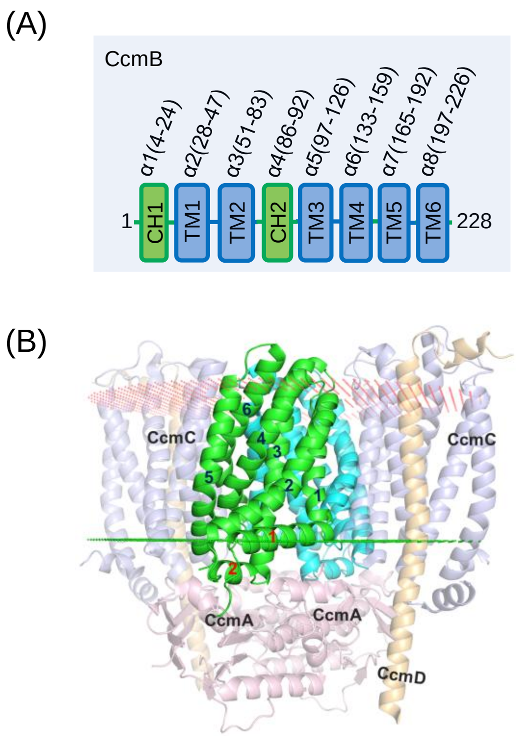

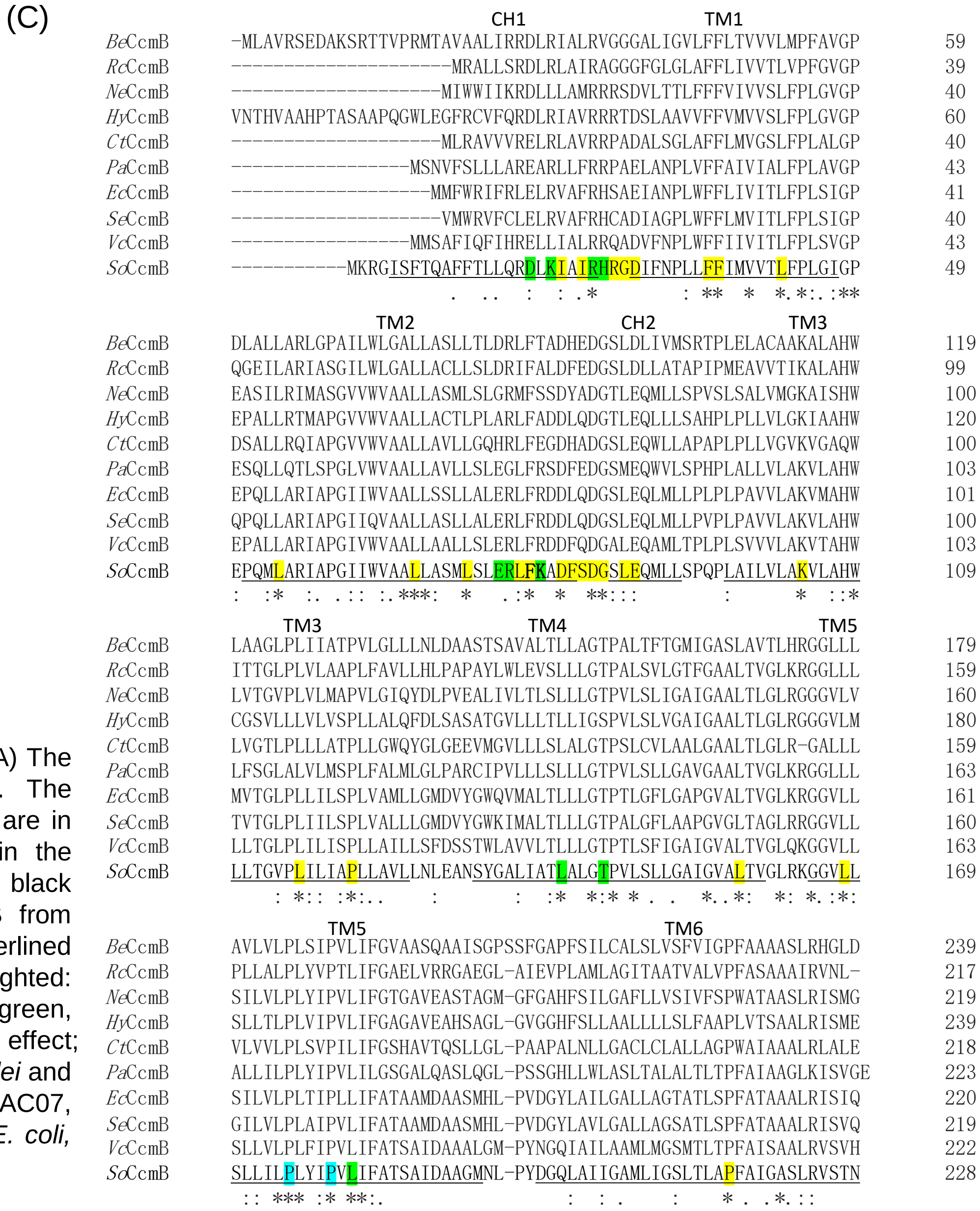

Figure S6. Sequence analysis of CcmB and its homologs. (A) The secondary structure elements of *S. oneidensis* CcmB. The cytoplasmic helices (CH) and transmembrane (TM) helices are in green and in blue, respectively. (B) The CcmB dimer in the $Ccm(ABCD)_2$ complex. TM and CH helices are numbered in black and red, respectively. (C) Sequence alignment of CcmB from representative bacteria hosting System I. α-helix are underlined and the residues subjected to mutational analysis are highlighted: in yellow, CcmB variants functioning the same as CcmB; in green, being able to work as CcmB for CCM but lack the detrimental effect; in blue, no activity at all. Bacteria include two α- *B. erythrophlei* and *R. capsulatus*, three β- *N. europaea, Hydrogenophaga* Sp. RAC07, and *C. terrigena*, and four γ-proteobacteria *P. aeruginosa*, *E. coli, Salmonella enterica*, *Vibrio cholerae*, and *S. oneidensis*.

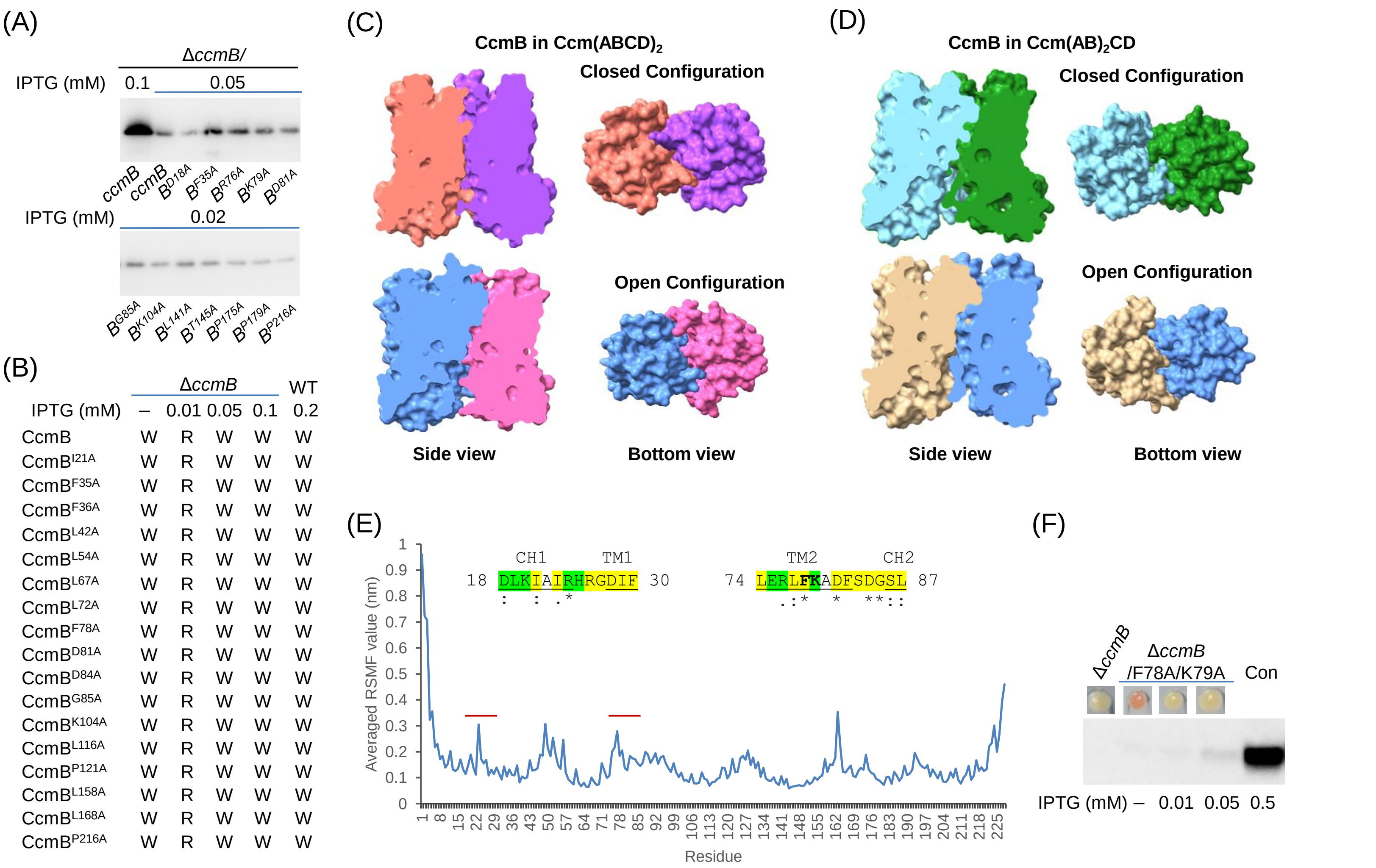

Figure S7. Characterization of CcmB. (A) Expression of CcmB variants examined by Western blot. (B) Functional analysis of CcmB variants. Shown are results from CcmB variants behaving the same as CcmB^{WT} in terms of CCM. (C, D) Heme pockets are not found in published cryo-EM structure of two CcmB dimers. The CcmB dimers are from the CcmABCD complexes in the forms of Ccm(ABCD)$_2$ (PDB: 8CE1) and Ccm(AB)$_2$CD (PDB: 8CEA and 7VFP). In each dimer, subunits are displayed in distinct colors. Upper and lower panels show the closed and open configurations. (E) RMSF analysis of CcmB. Two segments predicted to be important for heme entry are shown, with red lines showing the positions in CcmB. Residues subjected to mutational analysis are highlighted: in yellow, CcmB variants functioning the same as CcmB^{WT}; in green, being able to work as CcmB^{WT} for CCM but lack the detrimental effect. (F) Expression of CcmB$^{F78A/K79A}$ verified by Western blotting. Con, CcmB^{WT}.

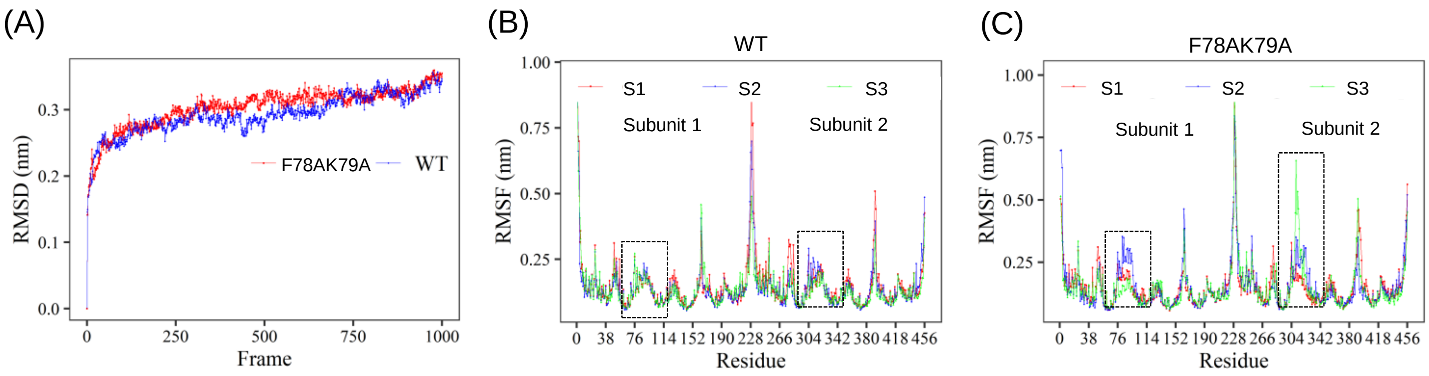

Figure S8. RMSD and RMSF analyses of CcmB and CcmB$^{F78A/K79A}$. (A) The average root-mean-square deviation (RMSD) of three independent simulations was calculated for both CcmB^{WT} and CcmB$^{F78A/K79A}$ dimers. Both WT and CcmB$^{F78A/K79A}$ CcmB dimers reached the stable status after approximately 200 ns. The RMSF of each independent simulation in CcmB^{WT} (B) and CcmB$^{F78A/K79A}$ (C). Residues 75-95 of CcmB$^{F78A/K79A}$ in both subunits (boxed areas) were unstable across all three simulations (S1, S2, S3). In contrast, these residues were found to be stable in CcmB^{WT}.

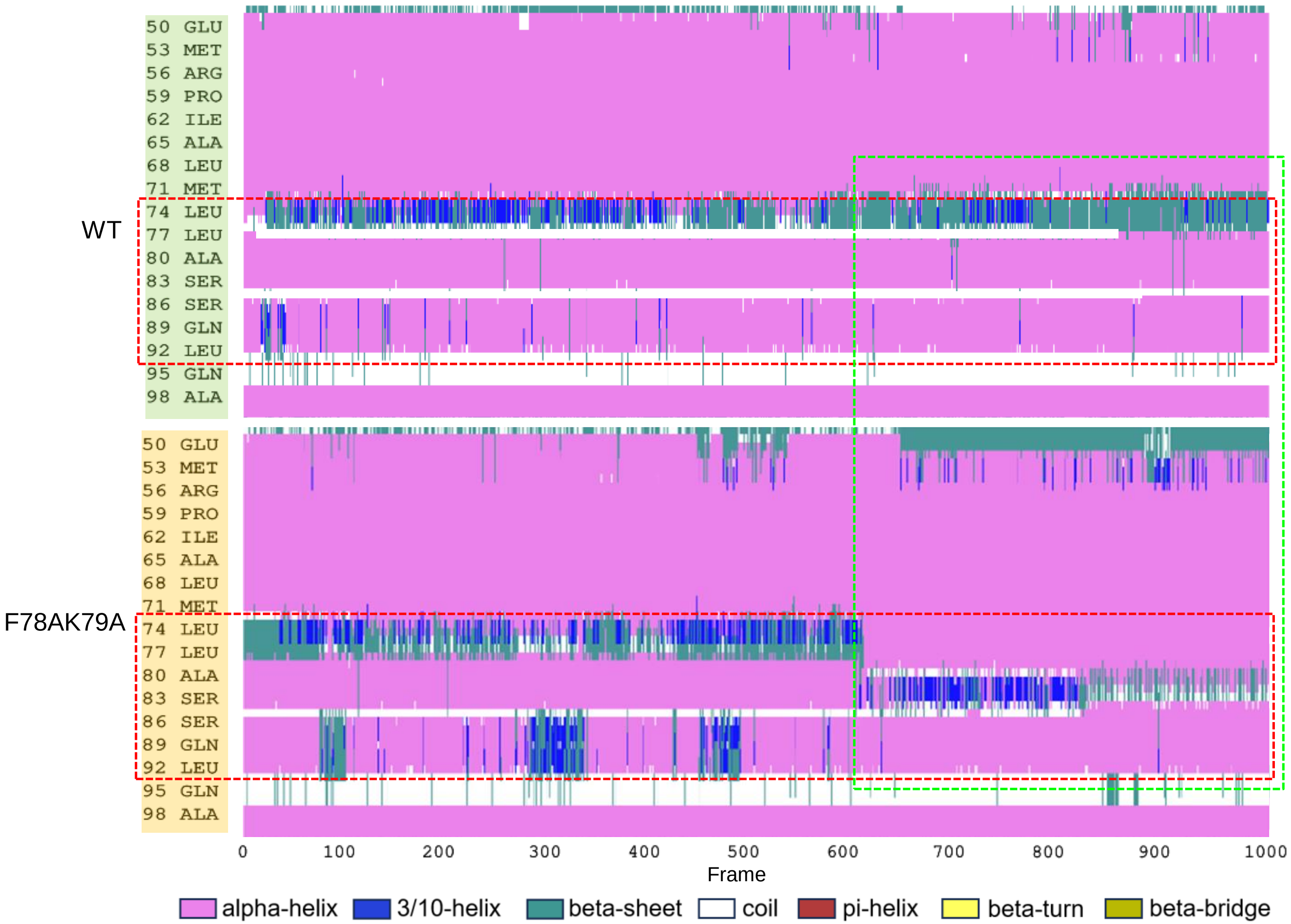

Figure S9. Dynamic secondary structure compositions of residues 50-100 in CcmB^{WT} and CcmB$^{F78A/K79A}$. The representative plots of Dictionary of Protein Secondary Structure (DSSP) for CcmB^{WT} and CcmB$^{F78A/K79A}$, which illustrate how the secondary structures of each residue change over time. Along y-axis, the residue numbers of two monomers (WT and the mutant in light green and light yellow backgrounds respectively); x-axis, frame number, with each frame representing 1 ns of simulation time. The predicted secondary structures of residues in a specific frame are presented in various colors (second structure types shown below). In monomers of ccmB^{WT} and ccmB$^{F78A/K79A}$, the DSSP of most residues under test are similar, except for residues 74-92. Within this region, the dynamic secondary structure compositions of ccmB^{WT} and ccmB$^{F78A/K79A}$ are clearly different (especially green boxed area), indicating that the mutation affects the stability of the secondary structure. Three independent analyses were carried out and the results were similar.

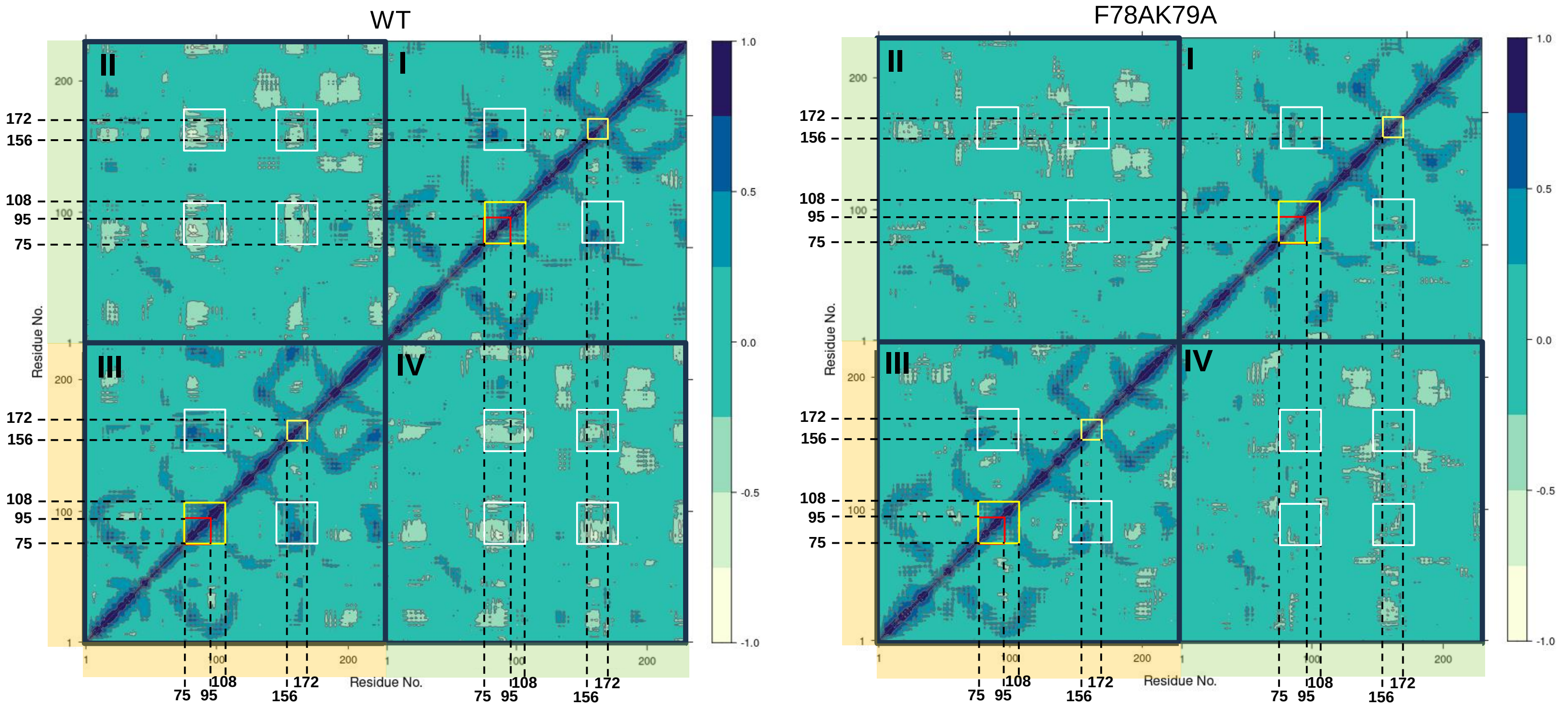

Figure S10. Residue cross correlation maps of $CcmB^{WT}$ and $CcmB^{F78A/K79A}$. These maps represent the time-correlated information between protein residues of $CcmB^{WT}$ and $CcmB^{F78A/K79A}$. Interaction of two monomers (with light green and light yellow margins) is shown. Each map is divided into four panels, where I and III represent the correlation within monomers, and II and IV represent the correlation between the two monomers. The degree of positive correlation (where two residues exhibit similar behavior) and negative correlation (where two residues exhibit opposite behavior) is indicated by the intensity of the dark blue and beige. In these maps, the white boxes mark the correlation regions between 75-108 and 156-172 residues, with the yellow and red boxes highlighting these two regions and the residues 75-95 (RMSF of this region is unstable in $ccmB^{F78A/K79A}$) respectively. Pairwise comparison of these regions marked by white boxes between $CcmB^{WT}$ and $CcmB^{F78A/K79A}$ reveals apparent differences in the motion correlation between residues 75-108 and 156-172, whether within monomer and between monomers, indicating that the mutation causes profound impacts on protein stability.

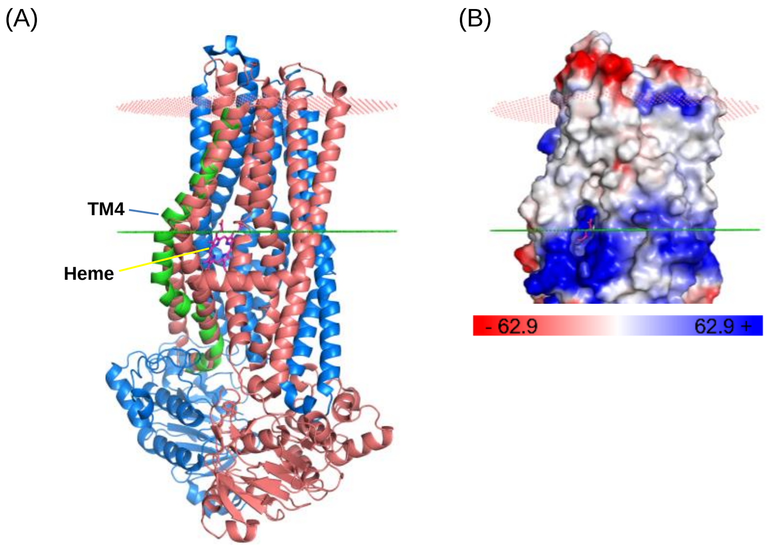

Figure S11. Structural features for heme entry. (A) CydDC (PDB 7ZDC) in the closed form. Two subunits are colored in pink and blue. The membrane-embedded segment of TM4 can move to the opened form (in green, PDB 7ZDB) and thus switches the entrance. The inner and outer leaflets of the membranes are shown in red lines and green dots respectively. (B) The vacuum electrostatics of fractional CydDC and the protein surface is visualized by assigning colors based on their charge distribution.

Table S1. Representative homologs of CcmB

Domain	Phylum	Class	Species	Length (a.a.)	BLASTp E-value	Operon	Activity[c]
Bacteria	Pseudomonadota	γ-proteobacteria	*Escherichia coli*	220	4.0e-78	ABCDE FGH	Yes
			Haemophilus influenza	221	3.0e-85	ABCDE FGHI	NT
			Pseudomonas aeruginosa	223	5.8e-65	ABCDE FGHI	Yes
		α-proteobacteria	*Rhodobacter capsulatus*	218	1.8e-53	ABCDE	Yes
			Bradyrhizobium erythrophlei	219	4.4e-52	ABCDE	NT
		β-proteobacteria	*Comamonas terrigena*	218	1.4e-51	ABCDE FGHI	Yes
			Nitrosomonas europaea	219	1.3e-65	ABCDE FGHI	NT
			Hydrogenophaga Sp. RAC07	239	3e-58	ABCDE FGHI	NT
	Acidobacteriota	Acidobacteriia	*Acidobacterium capsulatum*	224	4.2e-12	EAXB[a]	NT
	Chloroflexota	Ktedonobacteria	*Dictyobacter kobayashii*	229	5.9e-14	ABC	No
	Actinomycetota	Coriobacteriia	*Gordonibacter urolithinfaciens*	250	8.7e-11	ABC	NT
	Deinococcota	Deinococci	*Deinococcus radiodurans*	221	1.9e-15	AB	No
Archaea	Euryarchaeota	*Methanomicrobia*	*Methanococcoides burtonii*	224	5.0e-16	ABC	No
			Methanosarcina sp. MTP4	226	1.1e-13	ABC	NT
			Methanosarcina acetivorans	233	6.6e-13	ABC	No
Eukaryota	Streptophyta	*Magnoliopsida*	*Elaeis guineensis*	256	3.5e-57	BC[b]	Yes
			Hevea brasiliensis	489	2.2e-12	B	No
		Klebsormidiophyceae	*Klebsormidium nitens*	204	2.4e-11	B	NT
	Metazoa	Arthropoda	*Abscondita terminalis*	818	5.9e-36	B	NT
Viruses	Uroviricota	Caudoviricetes	Wolbachia phage WO	215	1.1e-11	B	No

[a] X represents non-*ccm* gene.
[b] the contig containing *ccmBC* genes could not be assembled into any of the chromosomes.
[c] NT, not tested.

Table S2. Strains and plasmids used in this study

Strain or plasmid	description	Source/reference
Strains		
E.coli		
DH5α	Host strain for plasmids	Lab stock
WM3064	Donor strain for conjugation; Δ*dapA*	W. Metcalf, UIUC
S. oneidensis		
MR-1	Wild type	Lab stock
HG0259	Δ*ccmE* derived from MR-1	(7)
HG0260	Δ*ccmD* derived from MR-1	(This study)
HG0261	Δ*ccmC* derived from MR-1	(This study)
HG0262	Δ*ccmB* derived from MR-1	(This study)
HG0263	Δ*ccmA* derived from MR-1	(This study)
HG0266	Δ*ccmF* derived from MR-1	(8)
HGCCMAB	Δ*ccmAB* derived from MR-1	(This study)
HGCCMBC	Δ*ccmBC* derived from MR-1	(This study)
HGCCMABC	Δ*ccmABC* derived from MR-1	(This study)
HGCCMABCD	Δ*ccmABCD* derived from MR-1	(This study)
HGCCMBCD	Δ*ccmBCD* derived from MR-1	(This study)
HGCCM	Δ*ccm* derived from MR-1	(This study)
HG1070	Δ*katB* derived from MR-1	(9)
HG3286-5	Δ*cydAB* derived from MR-1	(10)
Plasmids		
pHGM01	Ap^r Gm^r Cm^r suicide vector	(8)
pHG102	Constitutive active P*arcA* expression vector	(11)
pHGI01	Km^r, integrative *lacZ* reporter system	(12)
pHGEN-P*tac*	IPTG-inducible P*tac* expression vector	(13)
pHGEN-P*tac*-CcmB	inducible expression of CcmB	This study
pHGEN-P*tac*-CcmC	inducible expression of CcmC	This study
pHGEN-P*tac*-$CcmB^{His6}$	inducible expression of $CcmB^{His6}$	This study
pHGEN-P*tac*-CcmB-GFP	inducible expression of CcmB-GFP	This study
pHGEN-P*tac*-HO	inducible expression of *Synechocystis* HO	This study
pHG102-P*arcA*-smURFP	Constitutive expression of smURFP	This study
pHGEN-P*tac*-HrtBA	inducible expression of *C. diphtheriae* HrtBA	This study
pHGEN-P*tac*-HrtB	inducible expression of *C. diphtheriae* HrtB	This study
pHGEN-P*tac*-$CcmB^V$	inducible expression of various CcmB variants	This study
pHGEN-P*tac*-$CcmC^V$	inducible expression of various CcmC variants	This study
pHGEN-P*tac*-$CcmB^F$	inducible expression of foreign CcmB proteins	This study
pHGEN-P*tac*-HtpA-*GFP*	inducible expression of HtpA and GFP fusion proteins	This study
pHGEN-P*tac-FlhG-GFP*	inducible expression of FlhG and GFP fusion proteins	This study
pHGEN-P*tac-PetA-GFP*	inducible expression of PetA and GFP fusion proteins	This study
pHGI01-P*hemA*	promoter activity assays of *HemA*	(14)
pHGI01-P*hemC*	promoter activity assays of *HemC*	(14)
pHGI01-P*hemH1*	promoter activity assays of *HemH1*	(14)
pHGI01-P*hemH2*	promoter activity assays of *HemH2*	This study

References

1. Cook CE, Bergman MT, Cochrane G, Apweiler R, Birney E. The European Bioinformatics Institute in 2017: data coordination and integration. *Nucleic Acids Res* 2017;46:D21-D29.
2. Jumper J, Evans R, Pritzel A, Green T, Figurnov M, Ronneberger O, et al. Highly accurate protein structure prediction with AlphaFold. *Nature* 2021;596:583-589.
3. Oberg N, Zallot R, Gerlt JA. J.A. EFI-EST, EFI-GNT, and EFI-CGFP: Enzyme Function Initiative EFI. web resource for genomic enzymology tools. *J Mol Biol* 2023;435:168018.
4. Davidson AL, Dassa E, Orelle C, Chen J. Structure, function, and evolution of bacterial ATP-binding cassette systems. *Microbiol Mol Biol Rev* 2008;72:317-364.
5. Allen JWA, Harvat EM, Stevens JM, Ferguson SJ. A variant System I for cytochrome *c* biogenesis in archaea and some bacteria has a novel CcmE and no CcmH. *FEBS Lett* 2006;580:4827-4834.
6. Gupta D, Shalvarjian KE, Nayak DD. An Archaea-specific *c*-type cytochrome maturation machinery is crucial for methanogenesis in *Methanosarcina acetivorans*. *Elife* 2022;5:76970.
7. Wang W, Wang J, Feng X, Gao H. A common target of nitrite and nitric oxide for respiration inhibition in bacteria. *Inter J Mol Sci* 2022;23:13841.
8. Jin M, Jiang Y, Sun L, Yin J, Fu H, Wu G, et al. Unique organizational and functional features of the cytochrome *c* maturation system in *Shewanella oneidensis*. *PLoS ONE* 2013;8:e75610.
9. Jiang Y, Dong Y, Luo Q, Li N, Wu G, Gao H. Protection from oxidative stress relies mainly on derepression of OxyR-dependent KatB and Dps in *Shewanella oneidensis*. *J Bacteriol* 2014;196:445-458.
10. Chen H, Luo Q, Yin J, Gao T, Gao H. Evidence for the requirement of CydX in function but not assembly of the cytochrome *bd* oxidase in *Shewanella oneidensis*. *Biochim Biophys Acta* 2015;1850:318-328.
11. Wu L, Wang J, Tang P, Chen H, Gao H. Genetic and molecular characterization of flagellar assembly in *Shewanella oneidensis*. *PLoS ONE* 2011;6:e21479.
12. Fu H, Chen H, Wang J, Zhou G, Zhang H, Zhang L, et al.Crp-dependent cytochrome *bd* oxidase confers nitrite resistance to *Shewanella oneidensis*. *Environ Microbiol* 2013;15:2198-2212.
13. Meng Q, Liang H, Gao H. Roles of multiple KASIII homologues of *Shewanella oneidensis* in initiation of fatty acid synthesis and in cerulenin resistance. *Biochim Biophys Acta* 2018;1863:1153-1163.
14. Yin J, Meng Q, Fu H, Gao H. Reduced expression of cytochrome oxidases largely explains cAMP inhibition of aerobic growth in *Shewanella oneidensis*. *Sci Rep* 2016;6:24449.
